# Species Distribution Modeling of Killer Whales ( *Orcinus orca* ) in Australian Waters

**DOI:** 10.1002/ece3.71359

**Published:** 2025-07-03

**Authors:** Marissa J. Hutchings, Guido J. Parra, John A. Totterdell, Rebecca Wellard, David M. Donnelly, Jonathan Sandoval‐Castillo, Luciana Möller

**Affiliations:** ^1^ CEBEL, Cetacean Ecology, Behaviour and Evolution Laboratory College of Science and Engineering Flinders University Bedford Park South Australia Australia; ^2^ CETREC WA Cetacean Research Centre of Western Australia Esperance Western Australia Australia; ^3^ Project ORCA, Orca Research and Conservation Australia Kingsley Western Australia Australia; ^4^ Centre for Marine Science and Technology Curtin University Perth Western Australia Australia; ^5^ KWA, Killer Whales Australia Mornington Victoria Australia; ^6^ Dolphin Research Institute Hastings Victoria Australia; ^7^ MELFU, Molecular Ecology Laboratory College of Science and Engineering Flinders University Bedford Park South Australia Australia

**Keywords:** Australia, cetacean, dolphin, habitat suitability, killer whale, marine mammal, MaxEnt, orca, presence‐only, SDM

## Abstract

The killer whale ( *Orcinus orca* ) is a globally distributed apex predator. This species is represented by distinct ecotypes or forms, which are well documented in the Northern Hemisphere and Antarctica. However, less is known about killer whales in Australia. While research efforts have been made to study these animals, a broader understanding of their range and drivers of occurrence is lacking. In this study, we model the spatial distribution of killer whales in Australian waters to identify potential areas of habitat suitability and conservation priority. A total of 1310 sightings were compiled, of which 1115 were used alongside a suite of static and dynamic predictor variables to build presence‐only MaxEnt species distribution models (SDMs) in three separate study areas: southeast (SE), southwest (SW) and northwest (NW) Australia. The SDMs identified potential areas of habitat suitability both within and outside of known locations for killer whales in Australia. All three models returned good discriminative power between presence and background points. However, good predictive power was only suggested for the SE and NW. The importance of certain predictor variables indicated a preference for different environmental conditions, supporting the notion of at least two ecologically distinct groups. Killer whales frequenting the SE and SW preferred temperate waters, whereas those in the NW preferred tropical waters. This work greatly increases our understanding of killer whales in Australian waters and identifies potential areas of biological importance for management and monitoring. It also complements ongoing research into their genetics, feeding ecology, and diversification, showcases the utility of citizen science data, and informs the conservation of this species, which is still considered data deficient and remains to be adequately protected under Australian Government legislation.

## Graphical abstract

In this study, we model the spatial distribution of killer whales in Australian waters using MaxEnt. The presence‐only SDMs identified potential areas of habitat suitability and conservation priority both within and outside of known locations. Moreover, the importance of certain predictor variables indicated a preference for different environmental conditions, supporting the notion of at least two ecologically distinct groups of killer whales in Australia.

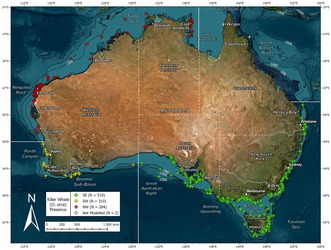

## 1 Introduction

Spatiotemporal structuring of the environment induces a similar organization of living organisms and their biological processes (Legendre and Fortin 1989). Species are thus aligned to their choice of habitat instead of being uniform or randomly distributed (Austin and Smith 1989). Some are restricted to a small number of locations in which they can effectively survive and reproduce (Guisan et al. 2006). Others can spread or move to enhance fitness and avoid unfavorable conditions (Holdo et al. 2009). This is most commonly driven by energetic gain, mate finding, and predator avoidance, but it can also be an adaptive response to environmental variation (Avgar et al. 2014). Ecological cues play an important role in the initiation and direction of animal migration (Bracis and Mueller 2017). However, natural or anthropogenic disturbance can mimic or mask these, leading to permanent range shifts and severe population fragmentation (Sha et al. 2008). For K‐selected species, which are long‐lived and slow‐maturing, such triggers impact distribution long before reproduction and survival (Ballance et al. 2006). Knowledge of where and when they occur is therefore critical to informing conservation management, particularly for keystone, rare, or endangered species challenged by the present‐day climate.

Modern technology has increased researchers' ability to assess patterns of species occurrence through space and time. However, there are still logistical challenges associated with locating, handling, and monitoring animals that are highly mobile and elusive (Elith et al. 2006). This is particularly true for the marine environment, where there are few physical barriers to movement and individuals often range freely. Furthermore, systematic surveys are costly and timely; thus, comprehensive datasets are lacking for many species (Kaschner et al. 2006). This has resulted in the distribution of most cetaceans being simply depicted as primary and secondary ranges on broad‐scale maps (MacLeod et al. 2008). These falsely portray that there is a similar probability of encountering a species over its entire geographic extent (Kaschner et al. 2011). Species distribution modeling (SDM) provides a solution to this problem. Records of occurrence are used to find patterns with large‐scale climatic factors in both known and unknown areas (Guisan and Zimmermann 2000). The relationships between these can then be used to infer drivers of occurrence and identify areas of habitat suitability. A range of SDM techniques exist, each with various strengths and limitations (Pasanisi et al. 2024). MaxEnt is a machine learning approach generally thought to provide the most accurate results for opportunistic datasets (Valavi et al. 2022). However, models must be adequately tuned with species‐specific parameters to balance overall complexity and fit (Radosavljevic et al. 2014).

The killer whale ( *Orcinus orca* ) is a globally distributed apex predator tolerant of a wide range of environmental conditions (Barrett‐Lennard et al. 2011). This species occurs throughout the world's oceans from the poles to the tropics in both coastal and pelagic settings. Their distribution is predominately governed by seasonal shifts in prey availability and large‐scale climatic factors (Baird 2000). A diverse diet and nomadic nature have allowed them to exploit various prey sources in a multitude of locations. In fact, matrilineal and philopatric social units filling novel environmental niches have promoted genome‐culture coevolution and led to the formation of distinct ecotypes or forms (Foote et al. 2016). Both sympatric and parapatric populations display differences in feeding ecology, morphology, social structure, bioacoustics, and genetics over fine spatial and temporal scales (De Bruyn et al. 2013). Recent work proposes species designations for the Northeast Pacific resident and transient killer whales (Morin et al. 2024). However, other studies suggest that more work is needed for those in other regions (Foote 2022; LeDuc et al. 2008). Due to taxonomic uncertainty, killer whales are still considered a single data‐deficient species (Reeves et al. 2017).

**FIGURE 1:**
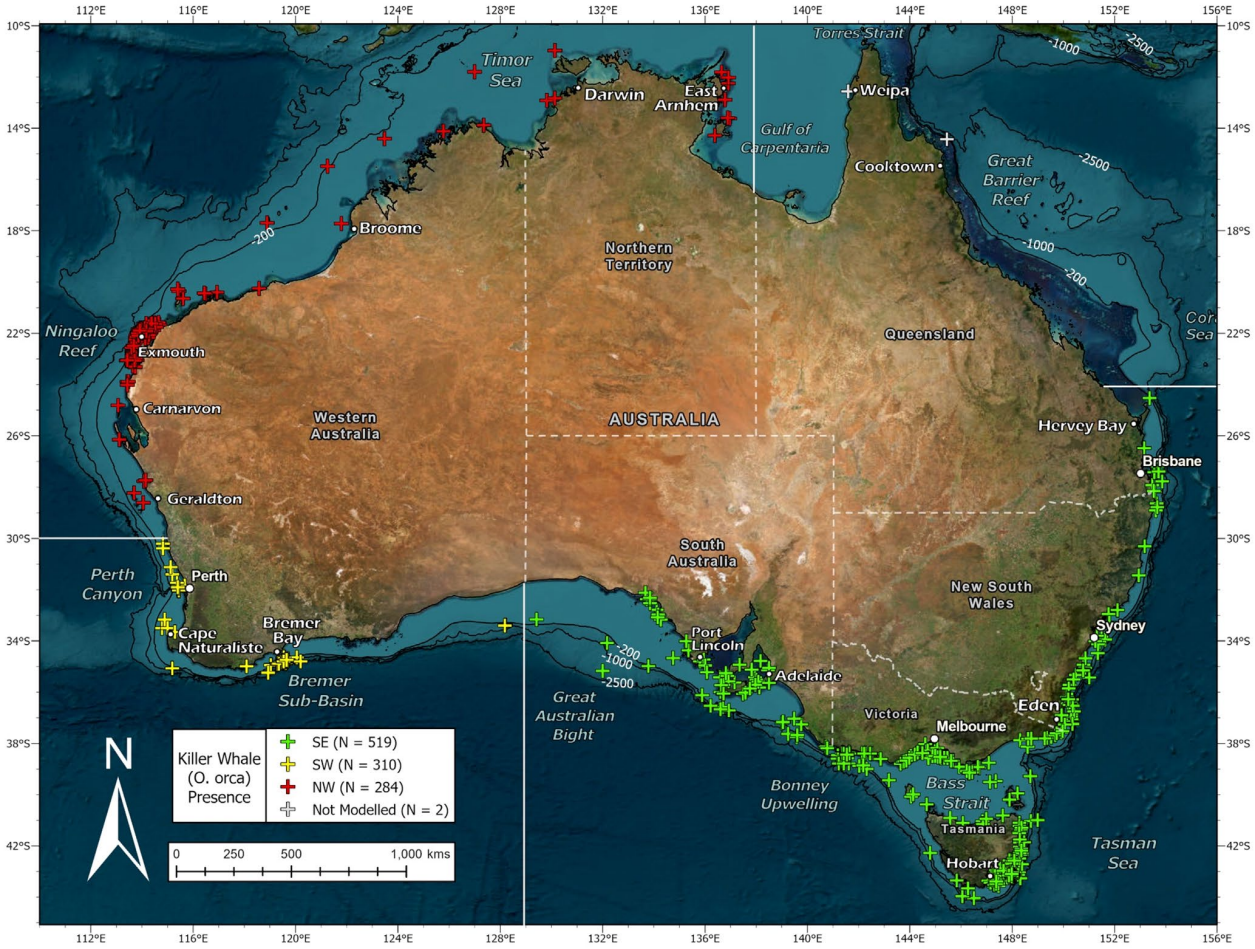
Map of killer whale (*Orcinus* orca) sightings compiled in Australian waters (*N* = 1115) showing which of those fell into each study area: southeast (SE, green), southwest (SW, yellow), northwest (NW, red) and not modelled (grey).

**FIGURE 2:**
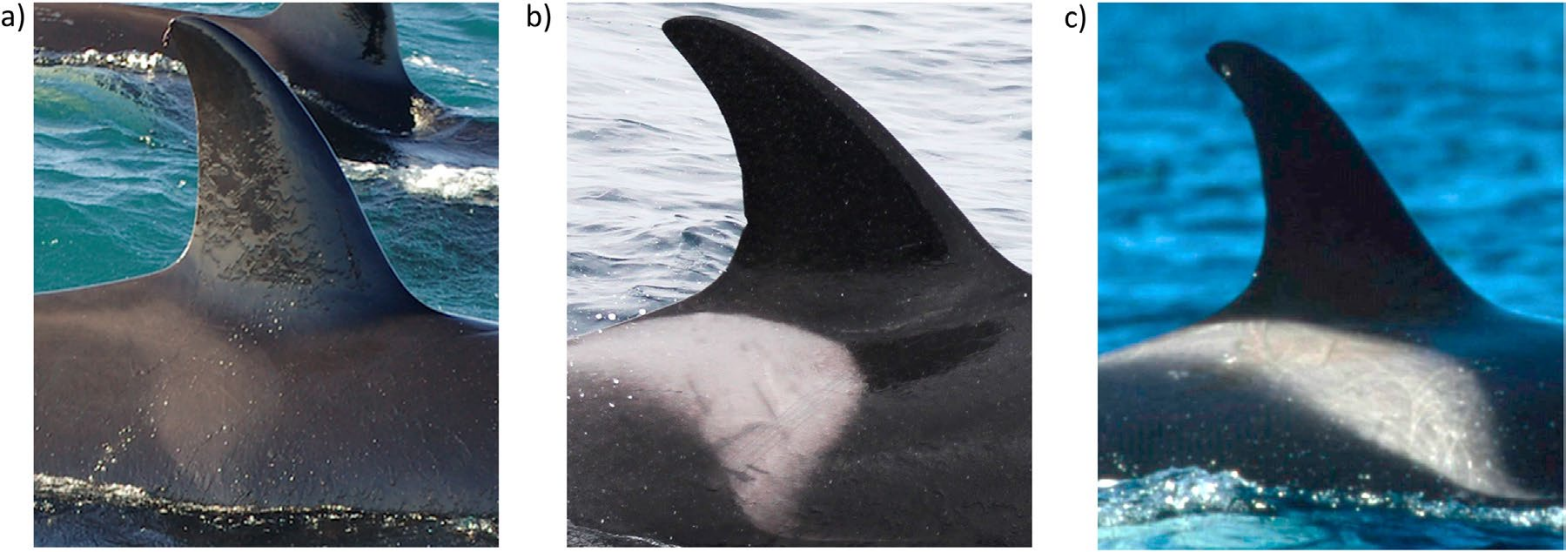
At least two forms of killer whales (*Orcinus* orca) may occur in Australian waters with varying prey choices, habitat preferences and phenotypic traits. In lower latitude regions including the (a) northwest (NW) study area, a tropical form exhibits darker and narrower saddle patches than a temperate form which has well contrasted pigmentation and can be sighted in both the (b) southwest (SW) and (c) southeast (SE) study areas.

Killer whales have been recorded year‐round in all coastal states and territories of Australia (Morrice 2004), but there are three locations where they are most commonly sighted. At least 79 individuals (17 of which were classed as Antarctic type C) have been identified in Australia's southeast (SE) (Donnelly et al. 2019), 200 or more (145 of which are catalogued) are known from the southwest (SW) Bremer Sub‐basin (Wellard and Erbe 2017) and another 52 (14 of which belong to a lesser‐known summer group) have been recorded at the northwest (NW) Ningaloo Reef (Totterdell and Wellard 2022) (Figure 1). Effort has been made to study the social structure (Wellard 2018), feeding ecology (Cieslak et al. 2021; Pitman et al. 2015; Totterdell et al. 2022; Wellard et al. 2016), bioacoustic repertoire (Wellard et al. 2015), population genetics (Reeves et al. 2022, 2023), and fine‐scale environmental drivers (Jones et al. 2019; Kämpf 2021; Salgado Kent et al. 2021) of these killer whales. Genetic research suggests some contact between them in the past, with those from the NW and SW study areas most disparate and those from the SW and SE study areas most alike (Reeves et al. 2022). However, there are currently no photo identification (photo‐ID) matches known to imply that individuals from any of these locations are still connected in present times (Donnelly et al. 2021). Based upon this fact, it is thought that there are at least three separate groups of killer whales occurring in Australian waters. More generally, there appears to be a tropical and temperate form with varying prey choices, habitat preferences, and phenotypic traits (Figure 2).

Knowledge of killer whale feeding ecology for Australia is limited, but they are thought to maintain a generalist diet. Those in the SE have been documented hunting other marine mammals, elasmobranchs, and teleosts (Morrice 2004). Killer whales have been sighted both on and off the continental shelf and are thought to span from Kangaroo Island in South Australia to Hervey Bay in Queensland (Donnelly, pers. comm.). They frequent areas of strong upwelling where there is increased productivity and an abundance of prey (Morrice 2004). Similarly, killer whales in the Bremer Sub‐basin are drawn to highly productive areas between the shelf edge and canyon heads (Kämpf 2021). These animals feed on beaked whales (*Ziphius* spp.) (Wellard et al. 2016), squid species, and pelagic fishes (Totterdell, pers. comm.), but also opportunistically pursue migrating pygmy blue whales ( *Balaenoptera musculus brevicauda* ) (Totterdell et al. 2022). Similarly, the winter group of Ningaloo Reef killer whales predate upon humpback whale calves ( *Megaptera novaeangliae* ) (Pitman et al. 2015). Anecdotal evidence suggests they have also attacked dwarf minke whales (*Balaenoptera acutorostrata sub*. sp.), spinner dolphins ( *Stenella longirostris* ) (Totterdell, pers. comm.) and Indo‐Pacific bottlenose dolphins ( *Tursiops aduncus* ) (Haughey et al. 2021). Individuals have been photo identified as far south as the Abrolhos Islands near Geraldton and as far north as the Rowley Shoals near Broome (Totterdell, pers. comm.). However, their transient nature implies that they likely exploit prey sources elsewhere.

Killer whales are also present outside of the forementioned locations. Sightings have occurred at lower latitudes in Western Australia, Queensland, and the Northern Territory. While poor imagery has so far prevented these animals from being matched to any known individuals, morphologically they resemble those from the Ningaloo Reef, having noticeably darker and narrower saddle patches (Mäkeläinen 2020). Similarly, killer whales have been sighted along the mid‐Western Australian coastline, specifically near Jurien Bay, along the Perth metropolitan area, behind Rottnest Island, and around Cape Naturaliste. Like those in the Bremer Sub‐basin, these animals have well‐contrasted pigmentation and feed upon other cetaceans, but they have not been matched with any known individuals (Wellard, pers. comm.). It is also unclear as to which group of animals a number of historic sightings along the South Australian coastline belong to. However, photo‐ID matches have been made between individuals sighted near Robe, located at the beginning of the Bonney Upwelling system, and the SE study area (Donnelly, pers. comm.).

Killer whales are widespread, abundant, and influential predators in Australian waters. Recent studies have established a knowledge base on these animals, and many individuals have been resighted across the years through photo‐ID. However, their occurrence has proven difficult to predict spatially, and a lack of information on the distribution of this species around the mainland and Tasmania currently hinders conservation management. This is a reason for concern, given that both natural and anthropogenic change are already impacting the movement of this species, along with many other cetaceans, globally (Kebke et al. 2022). In this study, we develop presence‐only MaxEnt SDMs for killer whales in Australian waters for conservational purposes. More specifically, we infer relationships between these animals and their environment, predict habitat suitability within three separate study areas, and project our results across a broader geographical extent around the continent. A secondary aim of this work was to explore the possibility of a tropical and temperate form of killer whale in Australian waters with contrasted habitat preferences that may help to distinguish them.

## 2 Methods

### 2.1 Species Occurrence Data

**FIGURE 3:**
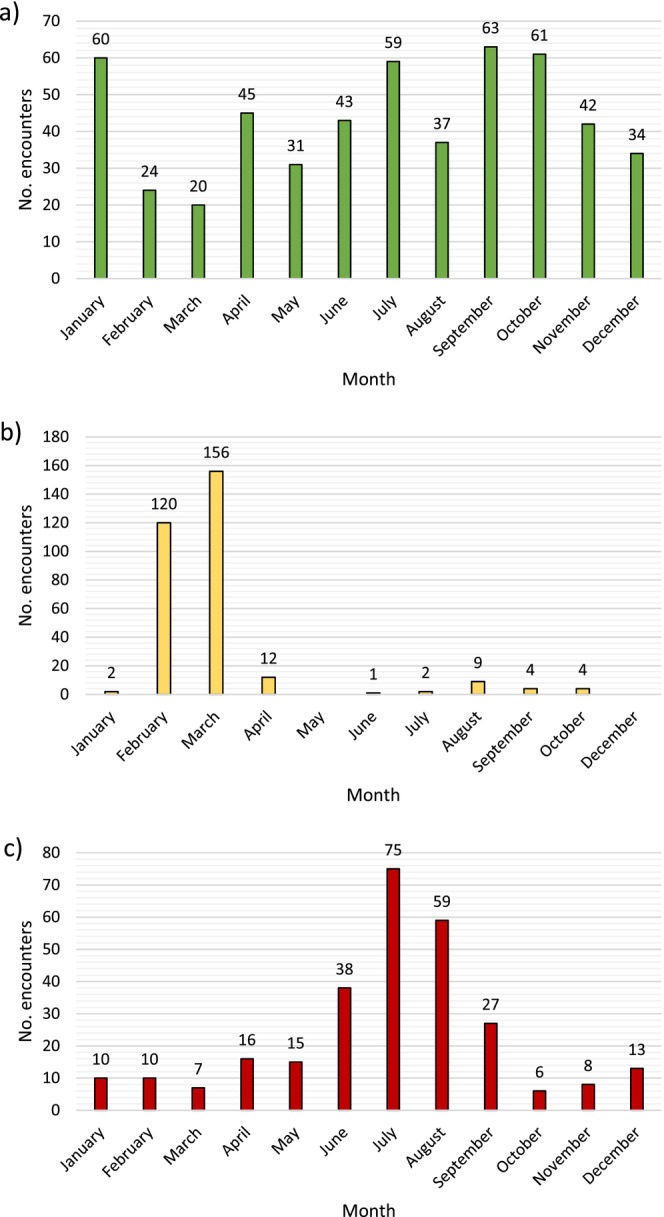
Monthly sightings of killer whales (*Orcinus* orca) in the (a) southeast (SE, green), (b) southwest (SW, yellow) and (c) northwest (NW, red) study areas.

A total of 1310 sightings of killer whales in Australian waters were compiled from a range of sources utilizing various sampling methods (Appendix S1). These occurred across all coastal states and territories and spanned 37 years from 1982 to 2023. Most sightings had been assigned exact coordinates with a GPS logger in the field, but in some cases georeferencing was used to estimate latitude and longitude from descriptions of generalized locations. Given opportunistic data can introduce bias, errors, and variation, a thorough quality check was performed to ensure reliability. 195 sightings, which mostly originated from citizen scientists or museum records, could not be verified for species identity by imagery or expert opinion and were thus filtered out. In ArcGIS Pro (v3.2) (Esri 2023), the remaining 1115 sightings were projected in decimal degrees (°) onto a base map using the WGS84 datum (Figure 1). This process was used to identify any positional errors or duplicate records. Sightings were not differentiated by ecotype as such designation does not exist for killer whales in Australian waters. However, given pre‐existing knowledge of killer whales in distinct geographical regions which are likely to have different habitat preferences, three separate study areas were defined for SDM, and one final model was presented for each. The number of sightings contained within these was greatest for the SE at 519, followed by the SW with 310 and then the NW which had 284. The three study areas encompassed all but two sightings in the filtered dataset, which both originated from northern Queensland. Despite substantial monitoring and research effort, the decision was made not to model this region due to a lack of sightings and overall understanding by researchers that killer whale presence here is sporadic. To assess temporal variation in species occurrence and survey effort, a bar chart of monthly sightings was created for each of the three study areas (Figure 3a–c).

### 2.2 Environmental Data

An initial list of 14 predictor variables was considered for SDM (Appendix S2). These included physical, biological, and chemical aspects of the marine environment which have been shown to influence the movement of cetaceans and other marine megafauna (Farmer et al. 2022; Kaschner et al. 2006; Willey et al. 2022). ArcGIS Pro was first employed to create a suite of four bathymetric and three distance‐based rasters that were deemed static in nature. Spatial controls of latitude and longitude were also made from scratch to capture any unexplained variance in the sightings. Data for five dynamic predictor variables was then downloaded from public online sources for the maximum time period available. To aid in interpreting any underlying temporal patterns, daily means of the latter were further subset with a custom Python script to create four parameters each of mean (Ave), maximum (Max), minimum (Min) and standard deviation (Std). This resulted in a total of 29 predictor variables for each study area. While these were classed as either static or dynamic in nature, it is important to note that the models themselves represent an average in environmental conditions across a longer time span. Temporal variation in the sightings and habitat preferences of killer whales was explored during preliminary analysis with seasonal and decadal SDMs. However, due to the small sample size of the datasets and thus poor performance of these models, results are not presented here, and species environmental relationships under differing climate or habitat changes remain to be validated. Similarly, modeling direct predator–prey relationships was outside the scope of this study given the inaccessibility and complexity of such datasets. The predictor variables used here are thus proxies of environmental conditions related to productivity and prey availability.

RStudio (v2023.12.1 + 402) (R Core Team 2023) was used to prepare concordant layers of the predictor variables for each study area through cropping to their respective extents, resampling to a 0.08° (9.2 kms) cell size and masking to either the 1000 m or 2500 m depth contour. This included another set of layers to encompass the combined extent of all three study areas and allow projection to the whole of Australia (Appendix S3). The predictor variables were then checked for multi‐collinearity by calculating their Pearson's correlation coefficients (R) and visualizing these in correlation matrices (Appendix S4a–c). Pairs that had an R value of greater than 0.75 or less than −0.75 were assessed in a stepwise manner and removed based on their correlations with other predictor variables (Fattahi et al. 2014). During preliminary analysis, predictor variables were then systematically varied across all study areas to assess their impact on model performance and generalization. The varSel and reduceVar functions of the R package ‘SDMtune’ (Vignali et al. 2020) were next consulted to see if they recommended alternative choices based on a default model. These functions build models iteratively to assess which predictor variable of each correlated pair maximizes permutation importance. However, the authors’ ecological reasoning behind each inclusion or exclusion was forefront when actioning these recommendations. A final suite of 14, 10, and 12 predictor variables were chosen from the initial list of 29 for SDM in the SE, SW, and NW, respectively (Table 1). These were subset and made into a RasterStack for SDM in each study area (Appendix S5a–c).

**TABLE 1:** Summary of the predictor variables retained and removed from the MaxEnt models built for the species distribution model (SDM) of killer whales (*Orcinus orca*) in Australian waters: southeast (SE), southwest (SW) and northwest (NW).

Study area	Retained	Removed
SE	D2L, D2RC, Depth, Slope, Aspect, VRM, ChlStd, SstAve, SstStd, SalStd, NCVMAx, NCVMin, ECVMax & ECVMin	D2CS, Lat, Long, ChlAveLog, ChlMaxLog, ChlMinLog, SstMax, SstMin, SalAve, SalMax, SalMin, NCVAve, NCVStd, ECVAve, ECVStd
SW	D2L, Depth, Lat, Aspect, VRM, ChlStd, SstMin, SalStd, NCVStd & ECVStd	D2CS, D2RC, Long, Slope, ChlAveLog, ChlMaxLog, ChlMinLog, SstAve, SstMax, SstStd, SalAve, SalMax, SalMin, NCVAve, NCVMax, NCVMin, ECVAve, ECVMax & ECVMin
NW	D2L, D2RC, Depth, Long, Slope, Aspect, ChlMinLog, SstMax, SstStd, SalStd, NCVMin & ECVMin	D2CS, Lat, VRM, ChlAveLog, ChlMaxLog, ChlStd, SstAve, SstMin, SalAve, SalMax, SalMin, NCVAve, NCVMax, NCVStd, ECVAve, ECVMax & ECVStd

*Note:* Predictor variables are abbreviated as follows: chlorophyll a concentration (Chl), distance to continental shelf break (D2CS), distance to land (D2L), distance to reef crest (D2RC), eastward current velocity (ECV), latitude (Lat), logged (Log), longitude (Long), maximum (Max), mean (Ave), minimum (Min), northward current velocity (NCV), salinity (Sal), sea surface temperature (Sst), standard deviation (Std), vector ruggedness measure (VRM).

### 2.3 Species Distribution Modeling

MaxEnt was employed to build presence‐only SDMs for killer whales in Australian waters for three separate study areas (Table 2). Additional machine learning approaches including random forest and boosted regression trees were also explored during preliminary analysis, but MaxEnt outperformed both in our case. To address sampling bias, a layer of target group survey effort was first created for each study area (Merow et al. 2013). Across the combined extent, a total of 4024 sightings of cetacean species were compiled and rasterised with the kernel density function in ArcGIS Pro. Three separate bias layers were then produced (Appendices S6a, S7a and S8a) with which survey effort could be perceived from and background point selection could be weighted for. Fewer background points were thus selected in areas of uncertainty due to sparse or absent input data. Using the randomPoints() function from the R package ‘dismo’ (Hijmans et al. 2011), 10,000 background points were randomly selected from both the SE and NW study areas following the recommendations of Elith et al. (2011). However, due to its smaller size, only 3000 background points were selected from the SW study area (Appendices S6b, S7b, and S8b). Preliminary analysis showed that this number provided enough environmental variation to train the model without sampling every available cell. Presence points were also thinned with the R package ‘spThin’ (Aiello‐Lammens et al. 2015) so that only one sighting per grid cell was considered by the model. While this process does reduce the number of presence points, it is considered the most effective way to eliminate autocorrelation and improve model accuracy (Stredulinsky et al., 2023). Spatial partitioning of both presence and background points was then performed using the R package ‘ENMeval’ (Muscarella et al. 2014) to create training and testing folds to build and evaluate the models for each study area (Appendices S6c,d, S7c,d, and S8c,d). Model tuning was next executed with the gridSearch() function from the R package ‘SDMtune’ (Vignali et al. 2020) to select the optimum regularization multiplier (R) and feature classes (FC) for each model. A total of 81 separate models were compared for each study area, and those which returned the highest true skill statistic (TSS) for the test data were selected.

**TABLE 2:** Summary of the MaxEnt models built for the species distribution model (SDM) of killer whales (*Orcinus orca*) in Australian waters: southeast (SE), southwest (SW) and northwest (NW).

Study area	Geographic extent	Depth contour used to mask	No. presence points (train/test)	No. background points (train/test)	Data partitioning method	No. predictor variables	Feature classes	Regularisation multiplier
SE	129°E, 155°E, −24°S, −45°S	2500 m	279 (223/56)	10,000 (8,000/2,000)	CheckerBoard2	14	LQH	3.5
SW	110°E, 129°E, −30°S, −36°S	2500 m	33 (26/7)	3000 (2400/600)	N ‐1 Jackknife	10	LHP	1.5
NW	110°E, 138°E, −10°S, −30°S	1000 m	95 (76/19)	10,000 (8,000/2,000)	CheckerBoard2	12	LQH	4.0

The final models were projected to their respective study areas, as well as to their combined extent around the continent, with clamping turned on to prevent overfitting and extrapolation (Radosavljevic et al. 2014). The cloglog output, which uses a scale of 0 to 1 as an index of habitat suitability, was chosen for its ease of interpretation (Merow et al. 2013). Models were evaluated with the threshold‐dependent true skill statistic (TSS) given this is a simple and intuitive metric largely robust to class imbalance (Allouche et al. 2006). TSS is calculated from sensitivity or the true positive rate (i.e., proportion of presences correctly classified as presences) and specificity or the false positive rate (i.e., proportion of background points incorrectly classified as presences) (Yoon and Lee 2023). TSS ranges from −1 to +1 where 1 indicates perfect agreement and below 0 no better than random (Yoon and Lee 2023). Values over 0.6 are considered good, 0.2 to 0.6 moderate, and below 0.2 poor (Komac et al. 2016). The influence of predictor variables was assessed through their permutation importance, jack‐knife testing results for TSS and univariate response curves (Appendix S9a–c). Expert knowledge of the species and environmental patterns was used to assess whether the predicted habitat suitability maps in each study area were ecologically realistic. Area under the curve (AUC) was not used as the primary evaluation metric for model performance due to the well‐recognized problems encountered with this approach (Jiménez and Soberón 2020). However, as a point of interest, the AUCs of each model were still reported in the Supporting Information (Appendix S10a–c). An ODMAP further detailing the SDM process was also prepared (Appendix S11) (Zurell et al. 2020).

## 3 Results

The results of each SDM will be presented by study area given that they represent killer whales in distinct regions of Australia with preferences for different environmental conditions.

**TABLE 3:** Summary of the MaxEnt model evaluations built for the species distribution model (SDM) of killer whales (*Orcinus orca*) in Australian waters: southeast (SE), southwest (SW) and northwest (NW). The train and test true skill statistic (TSS) as well as the difference between these are presented.

Study area	Train TSS	Test TSS	Difference
SE	0.6035	0.6147	−0.0112
SW	0.7840	0.6076	0.1764
NW	0.7466	0.7521	−0.0055

### 3.1 Southeast Australia

**FIGURE 4:**
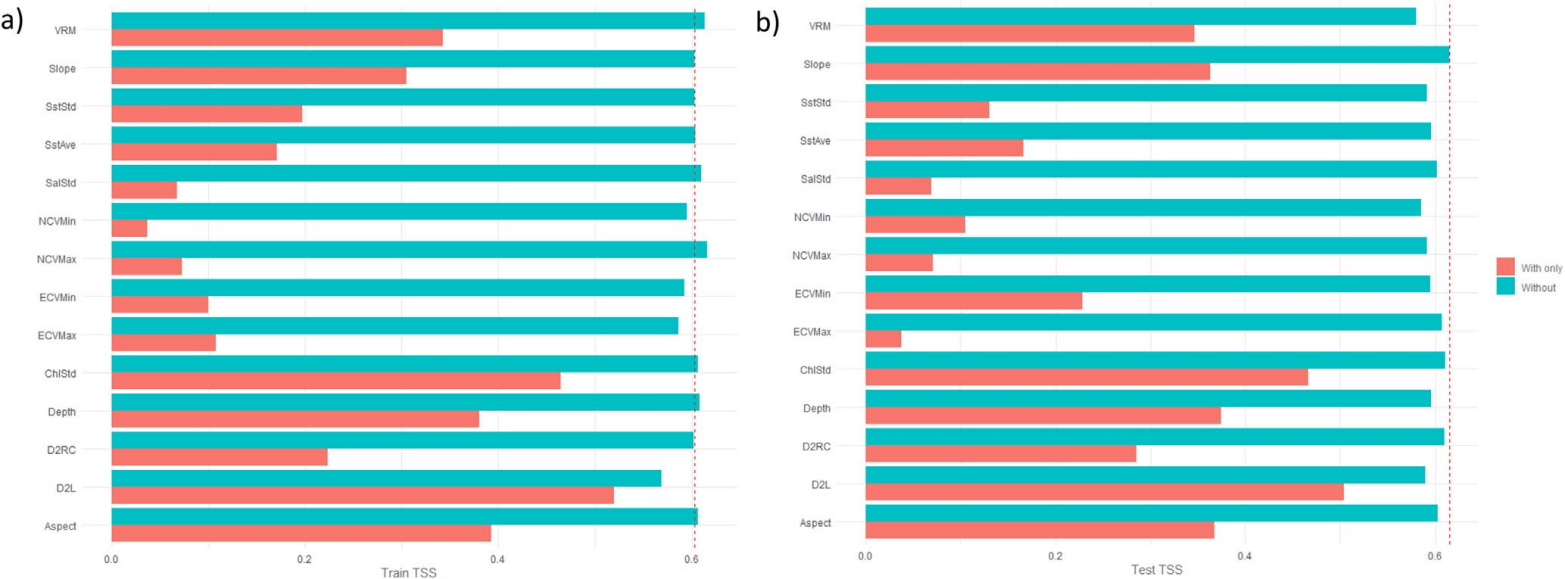
(a) Train true skill statistic (TSS) jack‐knifing and (b) test TSS jack‐knifing of predictor variables used by MaxEnt for the species distribution model (SDM) of killer whales (*Orcinus orca*) in Australian waters of the southeast (SE) study area. Predictor variables are abbreviated as follows: distance to land (D2L), distance to reef crest (D2RC), chlorophyll a concentration (Chl), sea surface temperature (Sst), northward current velocity (NCV), eastward current velocity (ECV), salinity (Sal), vector ruggedness measure (VRM), mean (Ave), maximum (Max), minimum (Min) and standard deviation (Std).

The ultimate MaxEnt settings determined through model tuning were FC = LQH and *R* = 3.5. This suggested a reasonably simple model with considerable smoothing. The final model was evaluated with a train TSS of 0.6035, test TSS of 0.6147, and a difference of −0.0112 (Table 3). This indicated that the model was moderately reliable at classifying presence and background points and had good discriminative power. The test data scored slightly higher than the train data, also suggesting good predictive power. According to permutation importance (Table 4), the top three predictor variables were distance to land (D2L) (30.9%), vector ruggedness measure (VRM) (20.0%), and depth (17.1%). However, in terms of both train (Figure 4a) and test TSS (Figure 4b) from the jackknife tests, the standard deviation of chlorophyll a concentration (ChlStd) obtained higher values than the latter two. Slope and aspect also often outcompeted VRM and depth. D2L remained the single most useful predictor variable when used in isolation to train and test the model across all accounts. The univariate response curves depicted either linear or hinged relationships between the presence and background points (Appendix S9a). These were negative or reverse for D2L, VRM, depth, ChlStd, aspect, and slope. Habitat suitability therefore decreased as values of these predictor variables increased, but there were no clear peaks. Preferred conditions for the killer whales in the SE study area were moderately deep waters (Depth: 0‐750 m) relatively close to land (D2L: 0°–0.75°) with high productivity (ChlStd: 1–5), a gentle slope (Slope: 0°–4°), low seafloor complexity (VRM: 0–0.002) and an eastward or southward dipping seabed (Aspect: 0°–225°).

**TABLE 4:** Permutation importance of predictor variables used by MaxEnt for the species distribution model (SDM) of killer whales (*Orcinus orca*) in Australian waters of the southeast (SE) study area.

Predictor variable	Permutation importance	Standard deviation
D2L	30.9	0.007
VRM	20.0	0.010
Depth	17.1	0.009
SstAve	9.8	0.005
NCVMin	5.8	0.004
Aspect	4.8	0.004
ECVMax	4.8	0.002
NCVMax	3.2	0.002
SalStd	1.4	0.002
ECVMin	0.8	0.001
SstStd	0.7	0.001
ChlStd	0.4	0.000
D2RC	0.2	0.000
Slope	0.1	0.001

*Note:* Predictor variables are abbreviated as follows: chlorophyll a concentration (Chl), distance to land (D2L), distance to reef crest (D2RC), eastward current velocity (ECV), maximum (Max), mean (Ave), minimum (Min), northward current velocity (NCV), salinity (Sal), sea surface temperature (Sst), standard deviation (Std), vector ruggedness measure (VRM).

**FIGURE 5:**
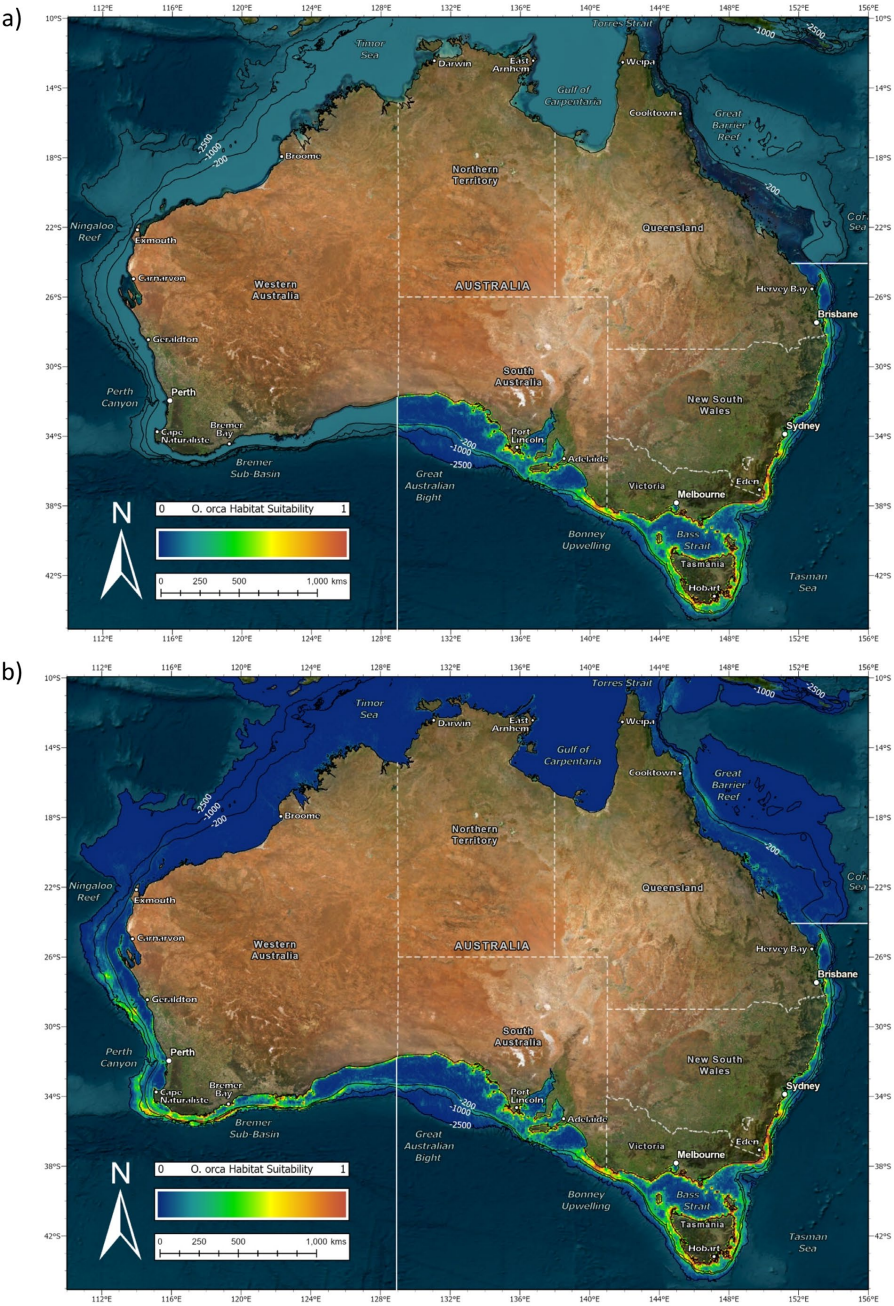
(a) MaxEnt habitat suitability map for the species distribution model (SDM) of killer whales (*Orcinus orca*) in Australian waters of the southeast (SE) study area and (b) projection of this to the whole of Australia.

The habitat suitability map for the SE study area (Figure 5a) showed moderate to high values along the majority of the lower New South Wales, Victorian, Tasmanian, and South Australian coastline. This peaked along the continental shelf break in the Bonney Upwelling region. However, several large gulfs were not considered suitable by the model. Similarly, habitat suitability decreased while moving up the New South Wales coastline and did not continue into Queensland waters. This was also the case when the SE model was projected to the whole of Australia (Figure 5b). It did not extend any higher than the northern boundary of the original study area near Hervey Bay. However, regions of high habitat suitability were predicted in the SW study area, including the Bremer Sub‐basin. Sporadic sections of moderate habitat suitability were also suggested along both the coastline and continental shelf as high as Carnarvon, but the model did not predict much further northward. It therefore deemed the Ningaloo Reef unsuitable for killer whales from the SE study area.

### 3.2 Southwest Australia

**FIGURE 6:**
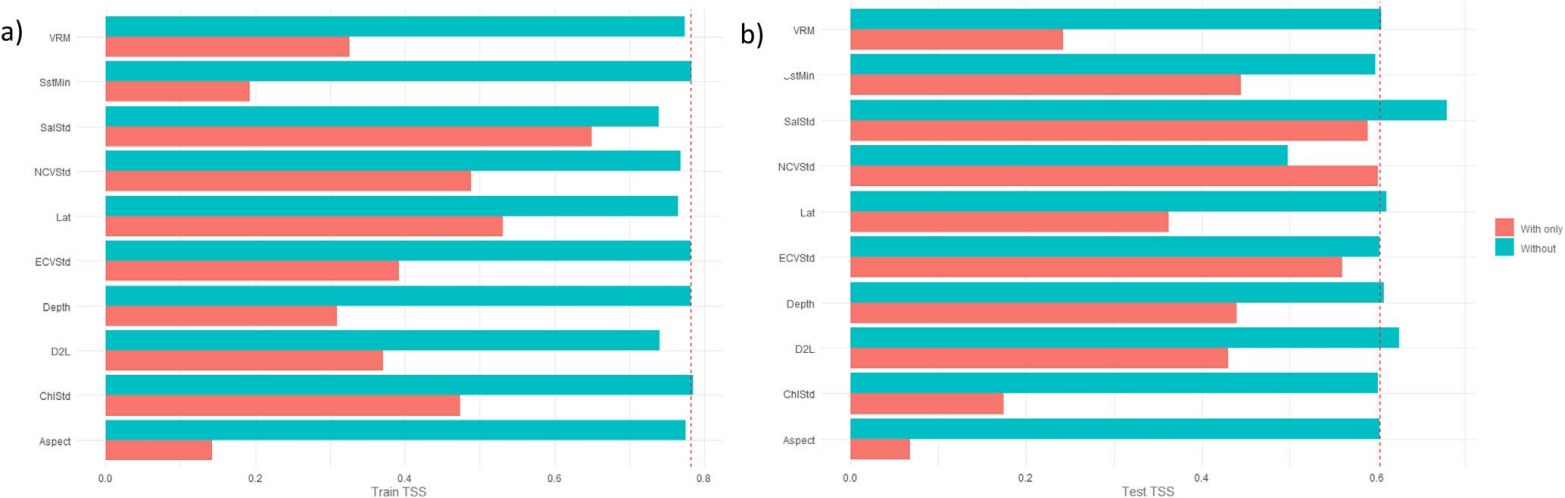
(a) Train true skill statistic (TSS) jack‐knifing and (b) test TSS jack‐knifing of predictor variables used by MaxEnt for the species distribution model (SDM) of killer whales (*Orcinus orca*) in Australian waters of the southwest (SW) study area. Predictor variables are abbreviated as follows: distance to land (D2L), latitude (Lat), chlorophyll a concentration (Chl), sea surface temperature (Sst), northward current velocity (NCV), eastward current velocity (ECV), salinity (Sal), vector ruggedness measure (VRM), minimum (Min) and standard deviation (Std).

The ultimate MaxEnt settings determined through model tuning were FC = LHP and *R* = 1.5. This suggested a reasonably complex model with slight smoothing. The final model was evaluated with a train TSS of 0.7840, test TSS of 0.6076, and a difference of 0.1764 (Table 3). This indicated that the model was highly reliable at classifying presence and background points and had good discriminative power. However, the test data scored much lower than the train data, thus suggesting poor predictive power. According to permutation importance (Table 5), the top three predictor variables were D2L (48.3%), the standard deviation of northward current velocity (NCVStd) (25.5%), and the standard deviation of salinity (SalStd) (20.3%). However, in terms of both train (Figure 6a) and test TSS (Figure 6b) from the jackknife tests, D2L obtained lower values than the majority of the other predictor variables. It was replaced by latitude in the training data and the standard deviation of eastern current velocity (ECVStd) in the testing data. NCVStd and SalStd remained the two most useful predictor variables when used in isolation to train and test the model. The univariate response curves depicted mostly parabolic relationships between the presence and background points (Appendix S9b). The distribution for NCVStd had one clear peak, whereas those of SalStd, latitude, and ECVStd were bimodal. D2L was represented by a reverse hinge. Habitat suitability therefore varied as values of these predictor variables increased. Preferred conditions for the killer whales in the SW study area were moderately shallow waters (Depth: 0‐500 m) close to land (D2L: 0°–0.5°) with variable currents (NCVStd: 0.025–0.150, ECVStd: 0.025–0.225) and salinity (SalStd: 0.125–0.375).

**TABLE 5:** Permutation importance of predictor variables used by MaxEnt for the species distribution model (SDM) of killer whales (*Orcinus orca*) in Australian waters of the southwest (SW) study area.

Predictor variable	Permutation importance	Standard deviation
D2L	48.3	0.046
NCVStd	25.5	0.027
SalStd	20.3	0.019
Depth	2.9	0.006
Lat	2.3	0.006
Aspect	0.4	0.002
VRM	0.2	0.001
ChlStd	0.1	0.001
ECVStd	0.0	0.000
SstMin	0.0	0.001

*Note:* Predictor variables are abbreviated as follows: chlorophyll a concentration (Chl), distance to land (D2L), eastward current velocity (ECV), latitude (Lat), minimum (Min), northward current velocity (NCV), salinity (Sal), sea surface temperature (Sst), standard deviation (Std), vector ruggedness measure (VRM).

**FIGURE 7:**
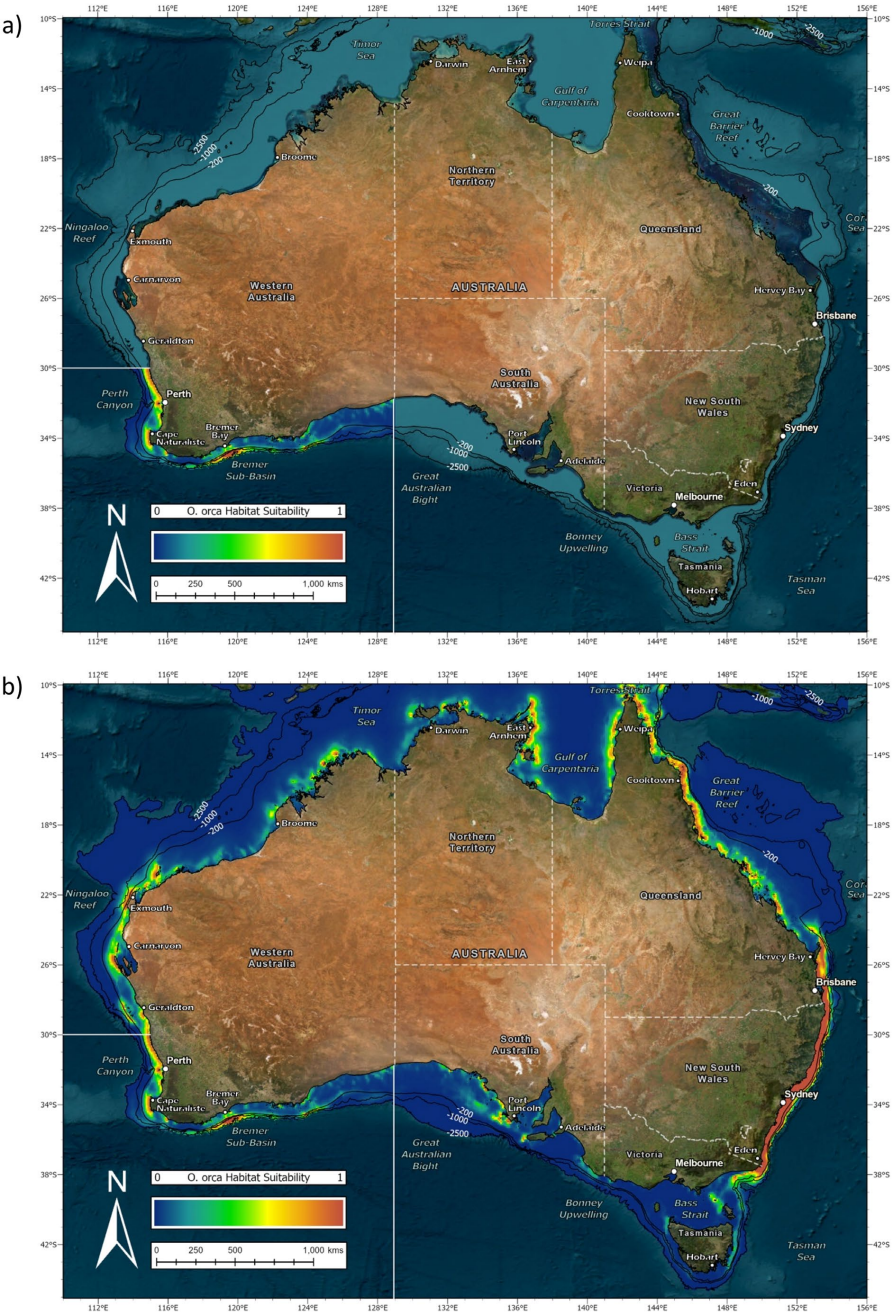
(a) MaxEnt habitat suitability map for the species distribution model (SDM) of killer whales (*Orcinus orca*) in Australian waters of the southwest (SW) study area and (b) projection of this to the whole of Australia.

The habitat suitability map for the SW study area (Figure 7a) showed high values in the Bremer Sub‐basin. This extended along the continental shelf break in both directions; however, no further east than Esperance. There was also suitable habitat predicted around Cape Naturaliste and along the Perth metropolitan coastline, the latter of which did not appear bounded by the northern extent of the SW study area. However, there was no suitable habitat predicted along the continental shelf break here. When projected to the whole of Australia (Figure 7b) the SW model predicted high habitat suitability in parts of both the SE and NW study areas. This included the entire coastline of New South Wales with smaller regions at the Ningaloo Reef and East Arnhem. It also suggested moderate habitat suitability along isolated pockets of the South Australian coastline, but did not show this for Victoria or Tasmania. Similarly, the SW model did not predict into the eastern Gulf of Carpentaria or the Great Barrier Reef.

### 3.3 Northwest Australia

**FIGURE 8:**
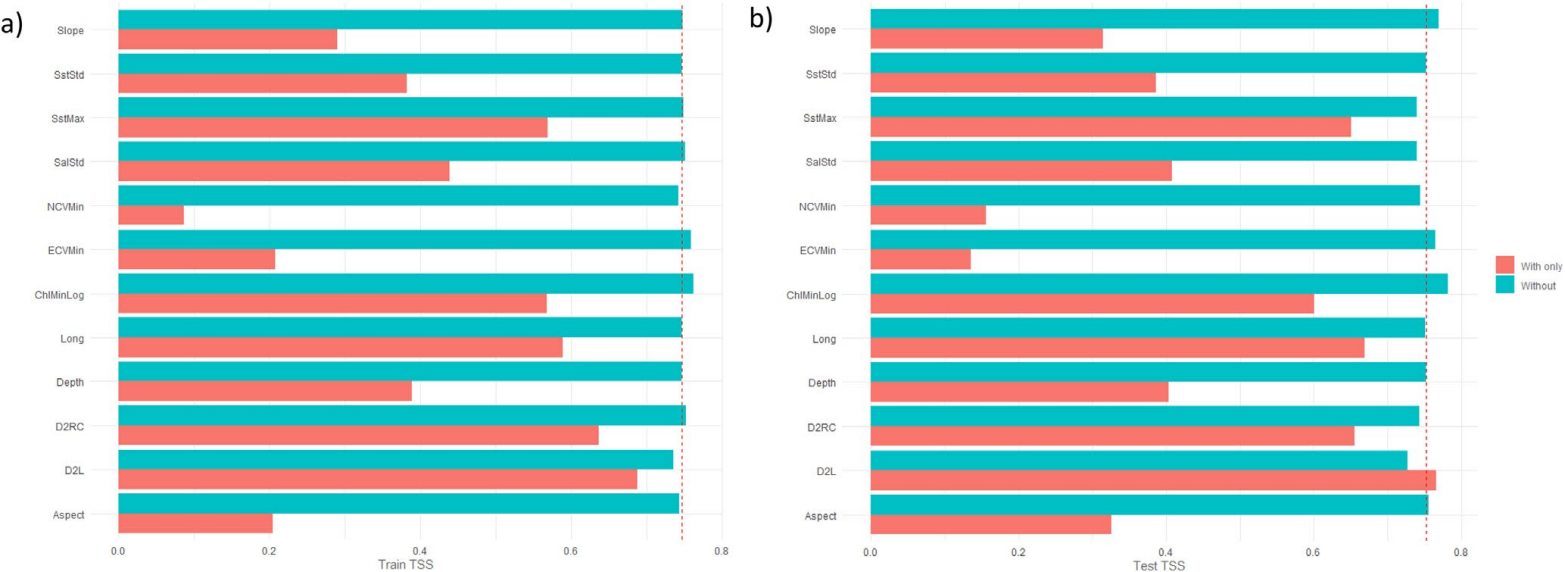
(a) Train true skill statistic (TSS) jack‐knifing and (b) test TSS jack‐knifing of predictor variables used by MaxEnt for the species distribution model (SDM) of killer whales (*Orcinus orca*) in Australian waters of the northwest (NW) study area. Predictor variables are abbreviated as follows: distance to land (D2L), distance to reef crest (D2RC), longitude (Long), chlorophyll a concentration (Chl), sea surface temperature (Sst), northward current velocity (NCV), eastward current velocity (ECV), salinity (Sal), maximum (Max), minimum (Min), standard deviation (Std) and logged (Log).

The ultimate MaxEnt settings determined through model tuning were FC = LQH and *R* = 4.0. This suggested a reasonably simple model with considerable smoothing. The final model was evaluated with a train TSS of 0.7466, test TSS of 0.7521, and difference of −0.0055 (Table 3). This indicated that the model was highly reliable at classifying presence and background points and had both good discriminative and predictive power. According to permutation importance (Table 6), the top three predictor variables were the minimum logged chlorophyll a concentration (ChlMinLog) (64.5%), maximum sea surface temperature (SSTMax) (18.3%), and SalStd (4.4%). However, in terms of both train (Figure 8a) and test TSS (Figure 8b) from the jackknife tests, they were always outcompeted by D2L, distance to reef crest (D2RC), and longitude. These three predictor variables, particularly D2L, therefore appeared to be the most useful when used in isolation to train and test the model. The univariate response curves depicted either quadratic or hinged relationships between the presence and background points (Appendix S9c). The distributions for ChlMinLog, SSTMax, and SalStd had one clear peak, whereas that of Long was bimodal. D2L and D2RC were represented by a reverse hinge. Habitat suitability therefore varied as values of these predictor variables increased. Preferred conditions for the killer whales in the NW study area were warm (SSTMax: 24°C–32°C) and productive (ChlMinLog: −2.5‐1.25) waters that were very close to land (D2L: 0°–0.25°) and coral reefs (D2RC: 0°–0.25°) with stable salinity (SalStd: 0–1.25).

**TABLE 6:** Permutation importance of predictor variables used by MaxEnt for the species distribution model (SDM) of killer whales (*Orcinus orca*) in Australian waters of the northwest (NW) study area.

Predictor variable	Permutation importance	Standard deviation
ChlMinLog	64.5	0.019
SstMax	18.3	0.005
SalStd	4.4	0.004
Slope	3.8	0.003
NCVMin	3.6	0.002
D2L	2.9	0.001
ECVMin	1.3	0.001
D2RC	1.0	0.004
Aspect	0.0	0.000
Depth	0.0	0.000
Long	0.0	0.000
SstStd	0.0	0.000

*Note:* Predictor variables are abbreviated as follows: chlorophyll a concentration (Chl), distance to land (D2L), distance to reef crest (D2RC), eastward current velocity (ECV), logged (Log), longitude (Long), maximum (Max), minimum (Min), northward current velocity (NCV), salinity (Sal), sea surface temperature (Sst), standard deviation (Std).

**FIGURE 9:**
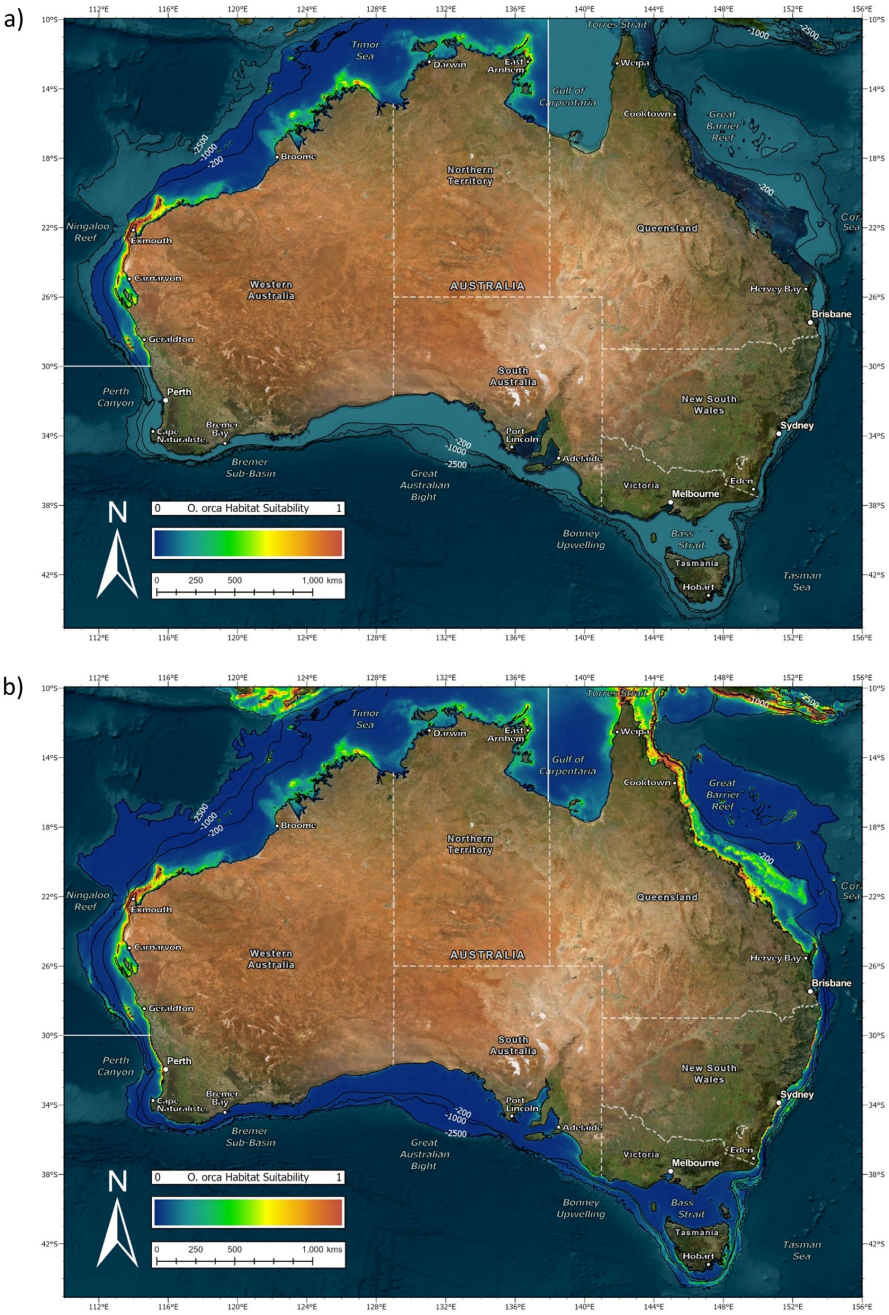
(a) MaxEnt habitat suitability map for the species distribution model (SDM) of killer whales (*Orcinus orca*) in Australian waters of the northwest (NW) study area and (b) projection of this to the whole of Australia.

The habitat suitability map for the NW study area (Figure 9a) showed high values along the Ningaloo Reef. This extended down the coast in a southerly direction toward Carnarvon and Geraldton. There was also suitable habitat predicted along the Kimberley's and East Arnhem Land as well as at the Montebello Islands and Abrolhos Islands. However, aside from Scott Reef where there was one sighting point, there was no suitable habitat predicted further offshore. When projected to the whole of Australia (Figure 9b), the NW model indicated further suitable habitat along the Perth metropolitan coastline. However, it did not predict around Cape Naturaliste or any further south. On the contrary, it did predict further northward into southern Indonesia and East Timor. This also extended across to Papua New Guinea, along the Great Barrier Reef and out to several islands in the Coral Sea. Moderate habitat suitability was suggested along some parts of the New South Wales coast, but only until approximately −36° S.

## 4 Discussion

Species–environment relationships have long been recognized as important aspects in biological, evolutionary, and conservation science (Guisan and Zimmermann 2000). However, due to the challenges associated with researching marine megafauna, there is a lack of knowledge regarding the distribution of many cetaceans. In this study, MaxEnt was successfully applied to predict the habitat suitability and drivers of occurrence for killer whales in three separate study areas: southeast (SE), southwest (SW) and northwest (NW) Australia. Each model was evaluated with a high TSS value indicating good discriminative power between presence and background points. Test TSS was higher than train TSS for both the SE and NW models, suggesting that they generalized well to independent data. This was reflected in reasonable projection outside of these study areas to a broader geographic extent. In contrast, the SW model exhibited lower test than train TSS, which may indicate overfitting and was evident in poor prediction outside of its study area. This variability in performance likely relates to differences in the sample size of each study area. Nonetheless, SDM identified highly suitable habitat for killer whales both within and outside of the currently known locations. Moreover, the importance of certain predictor variables indicated a preference for different environmental conditions. Killer whales frequenting the SE and SW preferred temperate waters, whereas those in the NW preferred tropical waters.

Sightings in the SE study area occurred during all months of the year, and the majority of these were relatively near to shore on the continental shelf. While not directly modeled, the width of the continental shelf likely plays a large role in the distribution of these killer whales. Habitat suitability was highest in locations where it narrowed toward the coastline. This included the lower corner of New South Wales, the east coast of Tasmania, and the Bonney upwelling region between Victoria and South Australia. Such locations are characterized by shelf‐incising canyons and strong hydrodynamics that concentrate plankton and attract an abundance of prey for killer whales (Middleton and Bye 2007). In contrast, habitat suitability decreased where the continental shelf widened across the central Great Australian Bight. This is a significant biogeographical feature that divides the distribution of many marine species (MacIntosh et al. 2018). Varied survey effort across the SE study area, which is greater along the more densely populated Victorian and New South Wales coastlines, likely contributed to this result. However, a number of historic sightings across South Australia confirm that killer whales do occasionally occur here. The SE model also predicted high habitat suitability throughout much of the SW study area. While no photo‐ID matches currently exist, shared genetic ancestry between these animals implies contact in the past (Reeves et al. 2022). Similarly, killer whales in the North Atlantic are believed to have diverged from Northeast Pacific ecotypes which made interoceanic crosses through the Canadian Arctic during periods of open ice (Moura et al. 2015).

It is interesting that northward and eastward current velocity (NCV and ECV) did not show up as important predictor variables for the SE model, seeing as there are several major currents flowing through this study area. The Leeuwin, Zeehan, and Flinders currents significantly influence productivity in the Great Southern Australian Coastal Upwelling System (Middleton and Bye 2007). There is also the East Australian Current that transports warm water southward along the New South Wales coastline. An increase in SST may limit the northern range of killer whales in the SE study area. In fact, photo ID suggests that most of these animals, except for some individuals that seem to prefer warmer waters, do not surpass Sydney (Donnelly, pers. comm.). However, Antarctic type B and C killer whales are known to perform six‐ to eight‐week migrations into warmer waters for skin molting (Durban and Pitman 2012). One individual completed a roundtrip of 9392 kms in just 42 days and experienced a 26°C change in SST (Pitman et al. 2020). Another travelled over 11,000 kms from the Ross Sea to northeast New Zealand, marking the longest known movement for this species to date (Pitman et al. 2020). While the killer whales considered here are not Antarctic type B or C, groups of these animals have been sighted in Australian waters on multiple occasions. This includes one account of an estimated 50 individuals travelling northward past Sydney (Donnelly et al. 2021). Killer whales from the SE study area also share genetic ancestry with those from New Zealand (Reeves et al. 2022), but it is unlikely that present‐day individuals venture off into the Pacific Ocean regularly, as there are no photo ID matches known between them.

The majority of sightings in the SW study area occurred in the Bremer Sub‐basin during February and March when platforms of opportunity and favorable weather conditions made data collection feasible. This is a place of high habitat heterogeneity and complex hydrodynamics situated on the continental shelf break, which is just 30 km from land (Exon et al. 2005). Thirty‐two other marine canyons lie within the broader SW study area between Cape Leeuwin and Esperance (Exon et al. 2005). The poleward flowing Leeuwin Current transports warm water into this region, creating the ultimate biophysical conditions to support a vast array of foraging marine species (Huang and Feng 2015). Killer whales feed at the canyon heads where there is flow‐induced upwelling and increased productivity (Kämpf 2021). Their relative density thus peaks approximately 40–50 km offshore in locations with low SST and high chlorophyll concentrations (Salgado Kent et al. 2021). Previous MaxEnt modeling in the Bremer Sub‐basin found water depth to be the most influential factor governing killer whale distribution with an optimum value of 1000 m (Jones et al. 2019). In comparison, it was ranked the fourth most important predictor variable for our SW model, and habitat suitability peaked much shallower at approximately 100 m. However, this result contradicts field observations and likely stems from the coarser spatial resolution used here. Spatial thinning also reduced the number of presence points, meaning there was less information for MaxEnt to consider.

As indicated by the habitat suitability map, killer whales likely frequent other parts of the SW study area. There have been no sightings offshore from Esperance, but killer whales from the Bremer Sub‐basin have been photo identified as far west as Cape Leeuwin (Wellard, pers. comm.) and satellite tracked to south of Albany at 118° E (Totterdell, pers. comm.). The SW model also predicted high habitat suitability around Cape Naturaliste and along the Perth metropolitan coastline. While no photo ID matches currently exist between the animals seen here and those in the Bremer Sub‐basin, a small number of individuals have been resighted at Rottnest Island since 2016 (Wellard, pers. comm.). This particular location is adjacent to aggregation areas and migratory routes for several other cetaceans, including pygmy blue whales, humpback whales, minke whales, and southern right whales ( *Eubalaena australis* ). Given attacks have been documented on the last three species mentioned, it is suggested that they may constitute an opportunistic prey source for killer whales in this region (Wellard, pers. comm.). As in the SE study area, Antarctic type B and C killer whales have also been observed off Western Australia (Donnelly et al. 2021). This includes at both the Bremer Sub‐basin and further north near Geraldton.

The majority of sightings in the NW study area occurred along the Ningaloo Reef, which spans 260 kms from Exmouth to Coral Bay and is a popular tourist destination during the austral winter (Spalding et al. 2001). Its proximity to the continental shelf, along with its latitudinal position, creates an area of unique hydrodynamics and high productivity (Taylor and Pearce 1999). Like the SW and SE study areas, SST in this region is influenced by the warm Leeuwin current, and these environmental conditions promote seasonal aggregations of marine megafauna (Sleeman et al. 2007). Most notably, humpback whales rest and calve in the Exmouth Gulf during their annual migration from Antarctica to the north of Broome (Irvine et al. 2018; Jenner et al. 2001). Killer whales take advantage of this by intercepting cow and calf pairs traveling both along the reef edge and inside the gulf. One group was satellite‐tracked for 1964 kms hunting between Exmouth and Carnarvon (Pitman et al. 2015), and four other individuals have been tagged making the same journey (Totterdell, pers. comm.). There is a second, more recently established humpback whale resting ground just south of this for which these animals have extended their range to encompass. Killer whales visiting the Ningaloo Reef may follow their prey even further south than this, with several individuals having been observed at the Abrolhos Islands near Geraldton. There is also evidence that killer whales off the Perth metropolitan coastline venture here (Wellard, pers. comm.).

Above the Ningaloo Reef, the continental shelf widens to accommodate several islands, atolls, and shoals (Collins 2011). While observer presence is low, killer whales are known to visit these offshore reef systems as well as similar habitats off the Kimberley coast and East Arnhem. Further north in the broader Indian Ocean, this species has been documented in Sri Lankan waters (Gemmell et al. 2015) and at the Maldives (Pro Divers Maldives 2023, November 8), but it is generally believed that their presence here is rare. Similarly, there are sporadic sightings from Papua New Guinea, the northern Great Barrier Reef, and various Coral Sea islands (Visser and Bonoccorso 2003). Theoretically, there is no reason why killer whales could not pass through the Torres Strait. However, shallow water, tidal extremes, and a mosaic of reefs would make for a challenging route. The southern equatorial current also bifurcates at the Queensland continental shelf, creating a natural boundary for many marine species (Wolanski 2017). While the NW model suggested good predictive power, it is extrapolating to a broader geographic area, and care must be taken when interpreting these results. For example, accessibility to the Great Barrier Reef lagoon is limited through channels that break up the outer edge, so it is unlikely killer whales occur here often.

While no ecotype has been described for killer whales in Australian waters, these results support the notion that both temperate and tropical forms occur here. The latter of which is a significant finding in and of itself considering a recent application of MaxEnt, used to model killer whale distribution on a global scale, only identified suitable habitat for this species around the southern half of the continent (Blanc and Martínez‐Rincón 2023). However, it is acknowledged that these authors may not have had access to sightings in northern Australia. The range of the temperate form is proposed to be below −24° S on the east coast of Australia. This marks the northernmost location in Queensland where killer whales in the SE study area have been photo identified and the northern boundary of the SE model which did not extend any higher than this when projected to the rest of the continent. It is more difficult to suggest a range for the temperate form in Western Australia, where predictive power of the SW model was considerably poorer. The southernmost extent of the tropical form is equally hard to define here. While killer whales visiting the Ningaloo Reef have been photo identified to approximately −30° S, which falls in line with the southern boundary of the NW model, it is likely that their range is expanding further south with the humpback whale migration. In fact, killer whales in the SW and NW study areas may well be sympatric in part of their range. The use of geographic regions as descriptors should therefore be treated with caution as it is unlikely that they align entirely with range. The presence of the tropical form in both the Northern Territory and Queensland is apparent, but where these animals originate from is yet to be confirmed with photo ID. In addition, more work is needed to explore genetic, phenotypic, and acoustic variation between the proposed forms. This will help to differentiate sightings and further resolve form‐specific habitat use (Eguiguren et al. 2019). It is also important to note that the SDM performed here was restricted to the 1000 m or 2500 m depth contour so no conclusions can be drawn regarding the distribution of killer whales within the broader Australian maritime jurisdiction.

All over the world, increased SSTs are pushing the distribution of marine mammals and their prey poleward (Kaschner et al. 2011). This would increase the range for the tropical form of killer whale in Australia, but the temperate form may have to move further offshore to persist. It is thus important to note that, although modeled uniformly here, shared environmental conditions may influence these forms differently. Similarly, while this species has adapted well to climate change in the past through expanding their niche, physiological tolerances, and prey preferences, this takes generations to achieve and may not be possible alongside the current unprecedented rate. In fact, specialized foraging of Northeast Pacific southern resident killer whales has already limited their ability to adapt to diminishing resources and led to population decline (Ford et al. 2010). Anthropogenic disturbance is another concern for this species globally, which may influence habitat preferences and suitability over time. Stressors such as commercial fishing, marine tourism, offshore drilling, and chemical pollutants are becoming increasingly prevalent in Australia (Morrice 2004). Concerningly, the Bremer Sub‐basin and Ningaloo Reef aggregations of killer whales are only partly protected by existing marine parks, leaving the majority of their habitat open to human use activities. Moreover, these fall under various government jurisdictions, which makes them challenging to monitor and impacts their overall effectiveness. Findings presented here can be used to define potentially biologically important areas, inform policymakers, and revise management plans.

Presence‐only SDM is not without its limitations, and the opportunistic nature of this dataset posed several challenges. Sampling bias is inherent in such cases, and while it was accounted for as best as possible with a layer of target group survey effort, it could not be eliminated entirely. For example, bar charts for the NW and SW study areas each show a clear peak in monthly sightings related to sampling bias. Moreover, the GPS coordinates of sightings were assigned on a first instance basis and thus only represent the beginning of an encounter. While this was necessary to avoid spatial autocorrelation, the models built here fail to capture the wider spread use of the study areas. Alternative modeling could be performed to address this with data derived from focal follows and measures of sampling effort. In addition, future work should strive to validate poorly surveyed regions with independent and systematic surveys to address projection uncertainty. This will subsequently help address whether certain predictor variables, such as distance to land, are acting as a proxy for survey effort. The SDMs would also benefit from an attempt to model direct predator–prey relationships once this data becomes available. For example, prey biomass can be compared with habitat suitability to further understand patterns in distribution (Davidson et al. 2023). Similarly, it would be worthwhile to further explore any temporal variation in the sightings and environmental preferences of killer whales with seasonal and decadal SDMs (Blanc and Martínez‐Rincón 2023). While it was not possible to do this here due to the small sample size of the datasets, the importance of incorporating multiple temporal and spatial scales to understand ecological adaptability cannot be overstated. This is particularly true for cetaceans with multi‐leveled and culturally segregated social structures (Vachon et al. 2022). Species are not in equilibrium with their environment; thus, SDMs must endeavour, where possible, to reflect this. These limitations should be addressed in future studies to improve understanding of killer whale distribution in Australian waters, but this will require dedicated sampling to increase the number of sightings across a larger geographical area and time span. Particularly given a limited number of presences versus background points, as seen for the SW study area, this can result in overfitting and inflation of evaluation metrics (Whitford et al. 2024). Aerial surveys, satellite tracking, and passive acoustic monitoring should be considered as potential means to do so. A digital platform for reporting sightings, such as Happywhale, could also be utilized to further encourage the contribution of other researchers, citizen scientists, and marine users (Cheeseman et al. 2017). Nonetheless, this work marks the first step forward in an exploratory, iterative, and adaptive process to inform future sampling design and improve model fit (Guisan et al. 2006).

Knowledge of species distribution is essential to the multidisciplinary field of ecology. This is particularly true, yet a challenging feat, for high trophic level organisms in the marine environment. This study provides substantial, valuable, and preliminary findings on the distribution of killer whales in Australian waters which can now be built upon in future analyses. It has increased our understanding of this species range and drivers of occurrence and identified areas of habitat suitability for targeted surveys and conservation priorities. It also supported the notion that there is a temperate and tropical form of killer whale in Australia and that the distribution of these animals is related to their environmental preferences. However, there is still much to learn about this species movements, feeding ecology, diversification, social structure, morphology, and genetics in the Southern Hemisphere. More research is needed to fill these knowledge gaps in order to build improved SDMs. A larger sightings dataset will enhance the power of such analyses, allowing finer spatial resolutions, exploration of temporal patterns, and modeling of predator–prey relationships. This will only be made possible by collaboration between researchers, citizen scientists, and marine users to improve the size and accessibility of datasets on both killer whales and their prey. These steps will be vital in ensuring that this species can be adequately managed in a changing environment.

## Author Contributions

**Marissa J. Hutchings:** conceptualization (equal), data curation (supporting), formal analysis (lead), funding acquisition (lead), investigation (equal), methodology (lead), project administration (equal), resources (equal), validation (equal), visualization (lead), writing – original draft (lead), writing – review and editing (equal). **Guido J. Parra:** conceptualization (equal), formal analysis (supporting), investigation (equal), methodology (supporting), project administration (equal), resources (equal), supervision (equal), validation (equal), writing – review and editing (equal). **John A. Totterdell:** conceptualization (supporting), data curation (lead), investigation (equal), resources (equal), validation (equal), writing – review and editing (equal). **Rebecca Wellard:** conceptualization (supporting), data curation (lead), investigation (equal), resources (equal), validation (equal), writing – review and editing (equal). **David M. Donnelly:** conceptualization (supporting), data curation (lead), investigation (equal), resources (equal), validation (equal), writing – review and editing (equal). **Jonathan Sandoval‐Castillo:** formal analysis (supporting), methodology (supporting), resources (equal), validation (equal), writing – review and editing (equal). **Luciana Möller:** conceptualization (equal), formal analysis (supporting), investigation (equal), methodology (supporting), project administration (equal), resources (equal), supervision (equal), validation (equal), writing – review and editing (equal).

## Conflicts of Interest

The authors declare no conflicts of interest.

## Supporting information


Appendix S1.

## Data Availability

The species occurrence data, spatial layers of predictor variables, and R code used to build the SDMs in this study have been deposited in figshare at https://figshare.com/s/a2a7c10ea620179ce91d.
