# Supplementary material for: Species Distribution Modeling of Killer Whales ( Orcinus orca ) in Australian Waters
Source: Ecol Evol. 2025 Jul 3;15(7):e71359. doi: 10.1002/ece3.71359 (PMC12223409; doi:10.1002/ece3.71359)
Supplement: Supplementary file 1 — Appendix S1. [file ECE3-15-e71359-s001.docx]

| **Source** | **Target species** | **Type of survey** | **General area** | **No. raw sightings** | **No. filtered sightings** | **No. incl. NW SDM** | **No. incl. SW SDM** | **No. incl.**  **SE SDM** |
| --- | --- | --- | --- | --- | --- | --- | --- | --- |
| Dave Donnelly  (Killer Whales Australia) | Killer whales | Vessel, land and aerial | Southeast Australia | 474 | 453 | 0 | 0 | 452 |
| Rebecca Wellard  (Project ORCA) | Killer whales | Vessel, land, aerial and acoustic | Bremer Sub-basin and Perth metropolitan coastline | 402 | 304 | 0 | 301 | 1 |
| John Totterdell (Cetacean Research Centre of WA) | Killer whales | Vessel and aerial | Ningaloo Reef and Bremer Sub-basin | 279 | 273 | 268 | 5 | 0 |
| Catherine Kemper (South Australian Museum) | All cetaceans | Vessel, land and aerial | South Australia | 107 | 58 | 1 | 3 | 54 |
| Atlas of Living Australia (Various) | Non-specific | Vessel, land and drone | Australia | 19 | 6 | 6 | 0 | 0 |
| Carol Palmer  (Charles Darwin University) | All cetaceans | Vessel, land and aerial | Northern Territory | 10 | 5 | 5 | 0 | 0 |
| Pete Gill  (Blue Whale Study) | Pygmy blue whales | Aerial | Great Australian Bight | 9 | 8 | 0 | 0 | 8 |
| Kirsten Rough (Australian Southern Bluefin Tuna Industry Association) | Southern Bluefin Tuna | Aerial | Great Australian Bight | 5 | 3 | 0 | 0 | 3 |
| Holly Raudino (Department of Parks and Wildlife WA) | Non-specific | Vessel, land, aerial, drone and acoustic | Western Australia | 2 | 2 | 2 | 0 | 0 |
| Chris Burton  (Western Whale Research) | Humpback and pygmy blue whales | Vessel, land and drone | Geographe Bay | 1 | 1 | 0 | 1 | 0 |
| Lyle Vail (Lizard Island Research Station) | Non-specific | Vessel, land and aerial | Northern Great Barrier Reef | 1 | 1 | 0 | 0 | 0 |
| Rebecca Dunlop  (University of Queensland) | Humpback whales | Vessel and land | Sunshine Coast | 1 | 1 | 0 | 0 | 1 |
|  |  |  | Total | 1310 | 1115 | 284 | 310 | 519 |

Appendix S1: Overview of sources and sample sizes of species occurrence data used in the SDM of killer whales (*Orcinus orca*) in Australian waters for each study area: southeast (SE), southwest (SW) and northwest (NW).

| **Predictor variable** | **Type** | **Source** | **Spatial resolution** | **Temporal extent** | **Ecological reasoning** | **Analysis notes** |
| --- | --- | --- | --- | --- | --- | --- |
| Sea surface temperature (SST) | Dynamic | Integrated Marine Observing System (IMOS) L3S Multi Sensor | 0.02° (2.2 km) | Daily mean (from 2012-2024) | SST drives prey aggregations | We used foundation day and night values then created layers of mean, maximum, minimum and standard deviation |
| Chlorophyll a concentration (Chl) | Dynamic | Integrated Marine Observing System (IMOS) OC3 model from MODIS | 0.01° (1.1 km) | Daily mean (from 2002-2023) | Chl acts as a proxy for prey biomass | We log transformed all values then created layers of mean, maximum, minimum and standard deviation |
| Northward current velocity (NCV) | Dynamic | Copernicus Marine Service Global Ocean Physics Reanalysis | 0.08° (9.2 km) | Daily mean (from 1993-2020) | NCV is related to upwelling and prey availability | We used the surface geostrophic V component to infer N/S directionality then created layers of mean, maximum, minimum and standard deviation |
| Eastward current velocity (ECV) | Dynamic | Copernicus Marine Service Global Ocean Physics Reanalysis | 0.08° (9.2 km) | Daily mean  (from 1993-2020) | ECV is related to upwelling and prey availability | We used the surface geostrophic U component to infer E/W directionality then created layers of mean, maximum, minimum and standard deviation |
| Salinity (Sal) | Dynamic | Copernicus Marine Service Global Ocean Physics Reanalysis | 0.08° (9.2 km) | Daily mean  (from 1993-2020) | Sal may represent physiological tolerences | We created layers of mean, maximum, minimum and standard deviation |
| Depth | Static | Geoscience Australia’s Bathymetry Grid | 0.0025° (277.5 m) | Collected in 2009 | Depth is related to upwelling and prey availability | We converted these from negative elevation values to positive depth values |
| Slope | Static | Derived from Depth | User specific | Constant | Slope is related to upwelling and prey availability | We used the terrain tool in RStudio |
| Aspect | Static | Derived from Slope | User specific | Constant | Aspect is related to upwelling and prey availability | We used the aspect tool in ArcGIS Pro |
| Vector ruggedness measure (VRM) | Static | Derived from Depth | User specific | Constant | VRM acts as a proxy for sub-marine canyons | We used the terrain ruggedness tool in ArcGIS Pro with neighbourhood size 3 |
| Distance to land (D2L) | Static | Derived from a shapefile of the Australian coatline obtained from the Australian Bureau of Statistics | User specific | Constant | D2L is related to depth, land-based prey and potentially survey effort | We used the distance accumulation tool in ArcGIS Pro |
| Distance to continental shelf break (D2CS) | Static | Derived from the 200m depth contour line obtained from eAtlas | User specific | Constant | D2CS is related to depth, upwelling and prey availability | We used the distance accumulation tool in ArcGIS Pro |
| Distance to reef crest (D2RC) | Static | Derived from a shapefile of coral reefs obtained from UNEP-WCMC et al. 2021 Global distribution of warm-water coral reeefs V4.1 | User specific | Constant | D2CS is related to depth, sheltered waters and prey availability | We used the distance accumulation tool in ArcGIS Pro |
| Latitude (Lat) | Static | Created from scratch | User specific | Constant | Lat acts as a spatial control | We used the RStudio package Raster |
| Longitude (Long) | Static | Created from scratch | User specific | Constant | Lat acts as a spatial control | We used the RStudio package Raster |

Appendix S2: The initial list of predictor variables considered in the SDMs of killer whales (*Orcinus orca*) in Australian waters.

Appendix S3: Spatial layers of all the predictor variables used in the SDM of killer whales (*Orcinus orca*) in Australian waters.


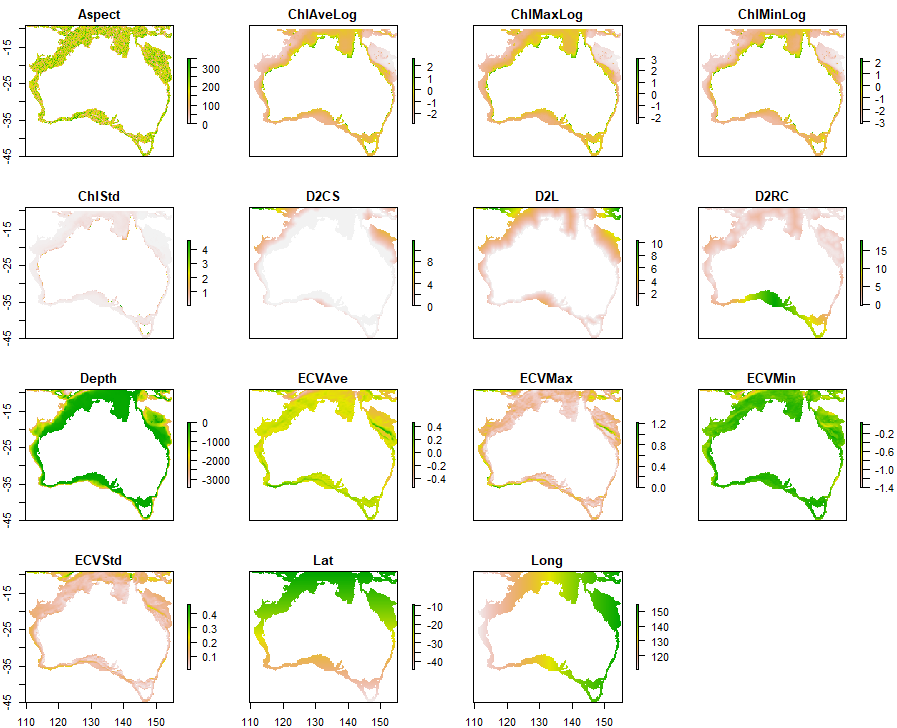

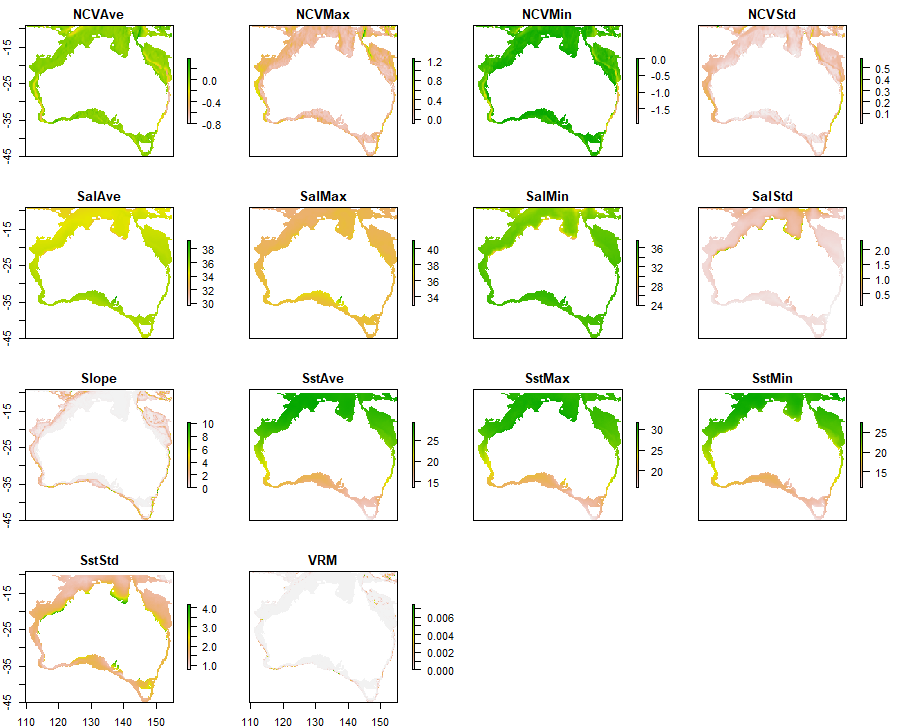

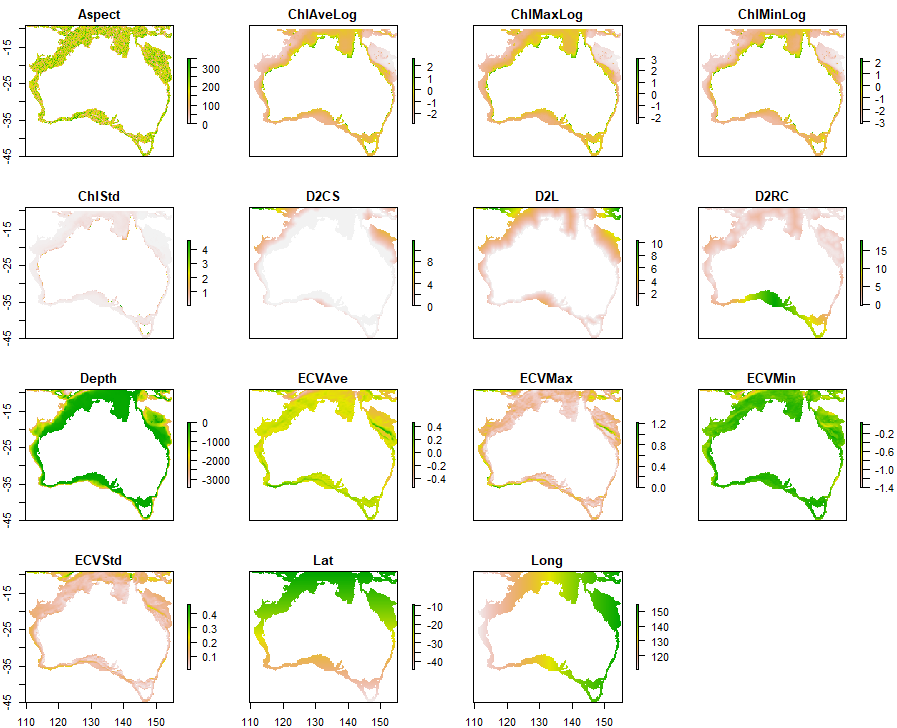

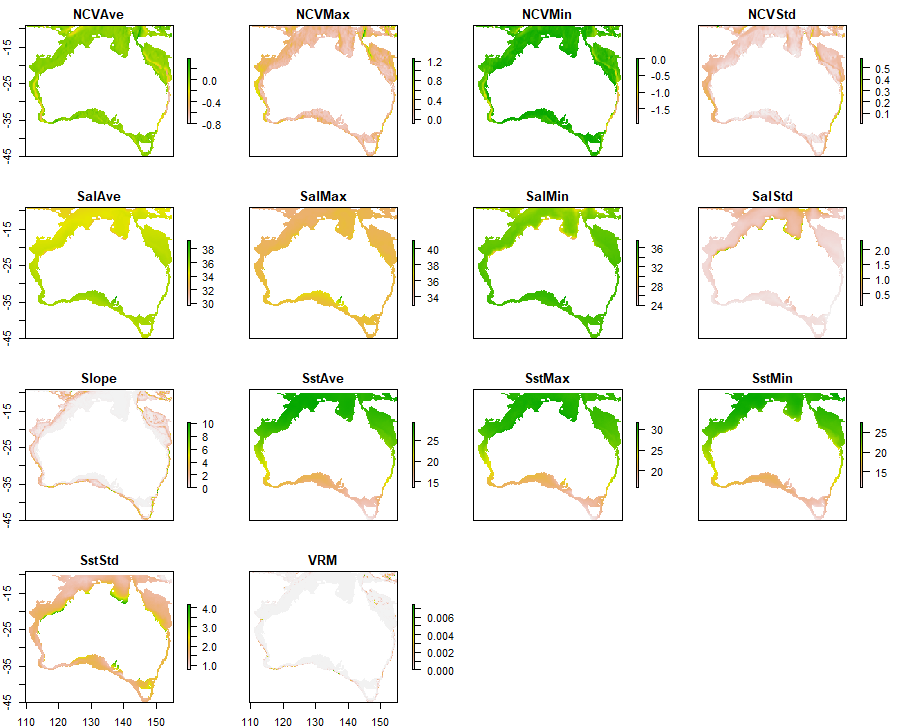

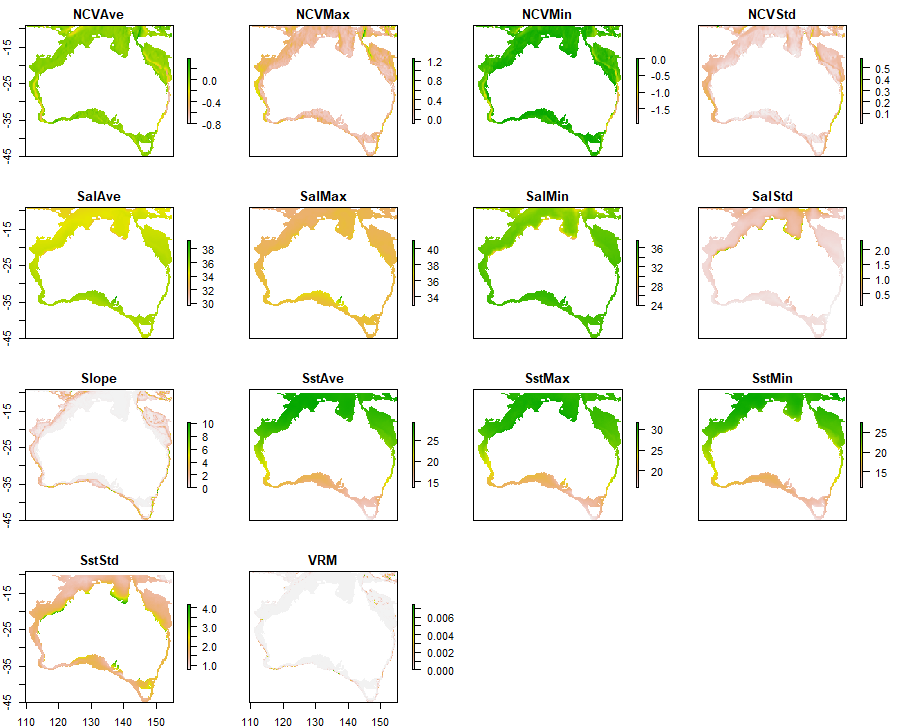

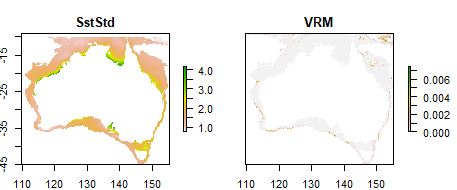


Appendix S4: a) Correlation matrix of all the predictor variables considered in the SDM of killer whales (*Orcinus orca*) in Australian waters: a) for the southeast (SE) study area, b) for the southwest (SW) study area and c) for the northwest (NW) study area. Values are displayed for those which had R > 0.75 (red) or < -0.75 (blue).


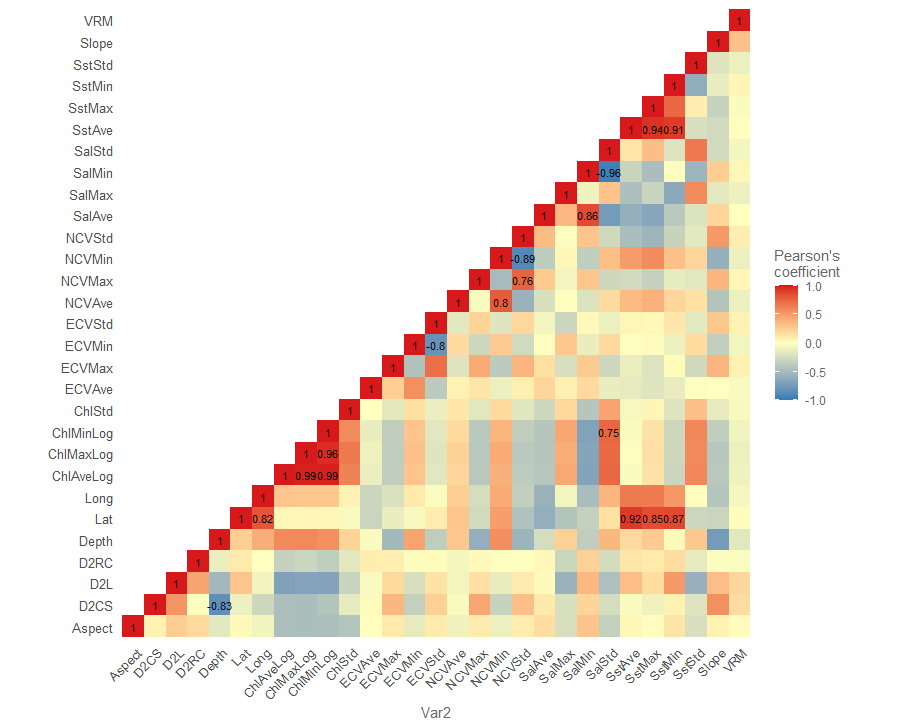

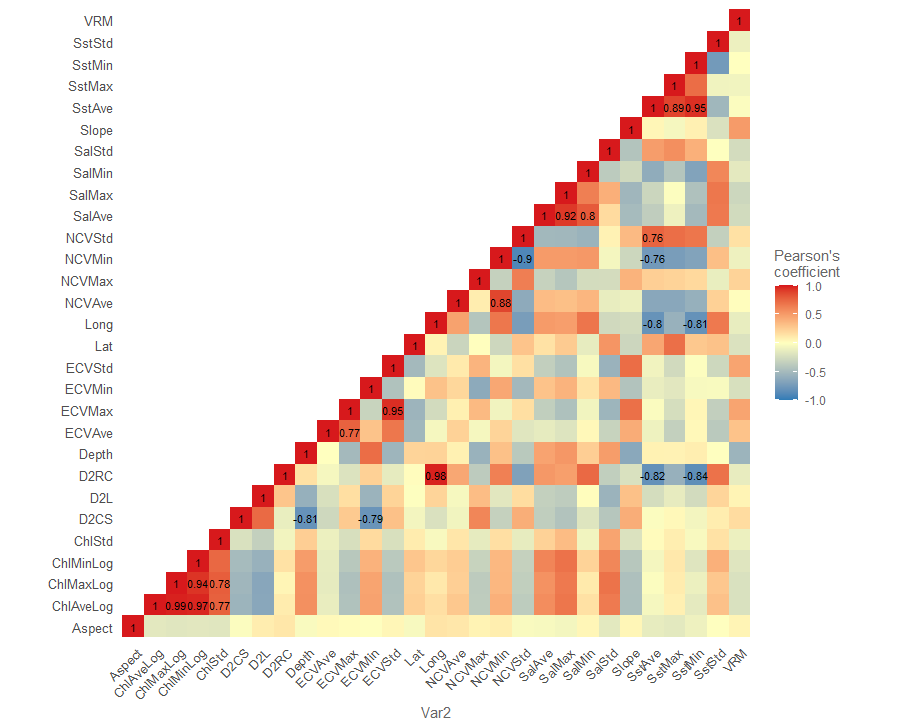

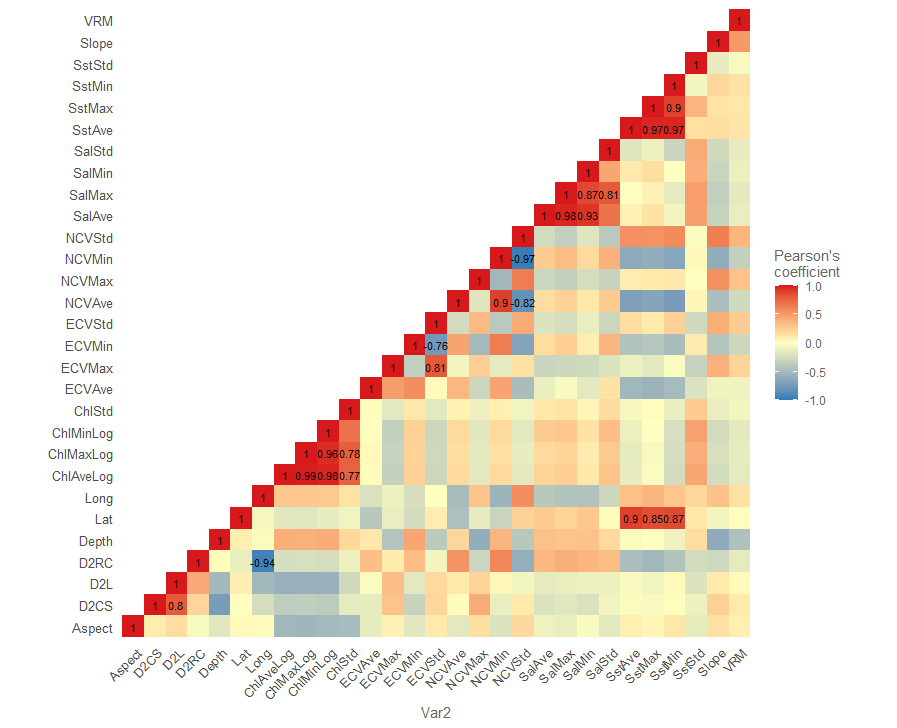


c)

b)

a)


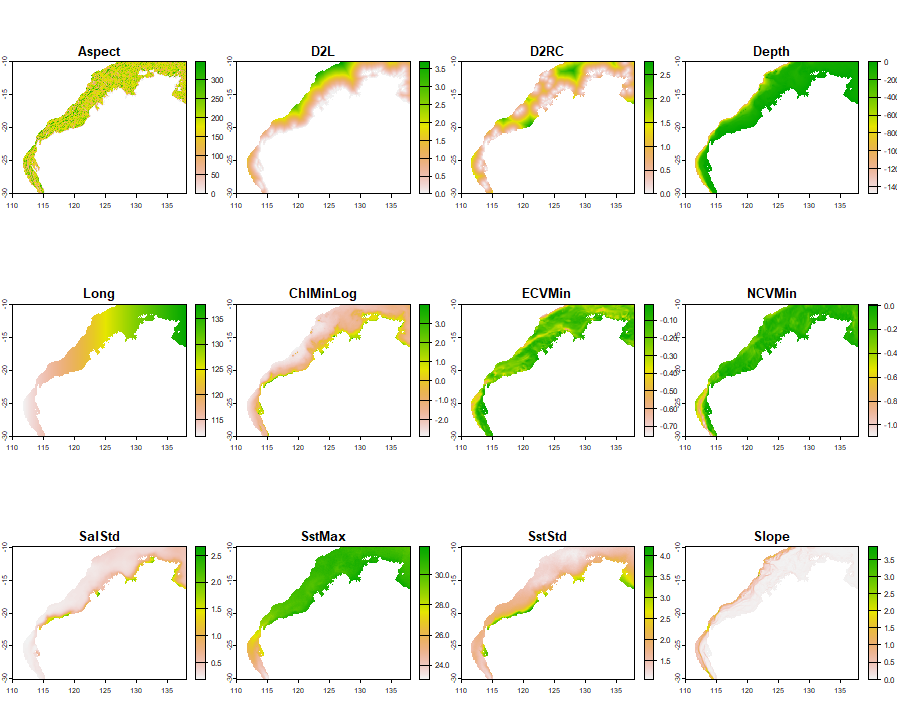
Appendix S5: a) The final suite of spatial layers chosen for the SDM of killer whales (*Orcinus orca*) in Australian waters: a) of the southeast (SE) study area, b) of the southwest (SW) study area and c) of the northwest (NW) study area.


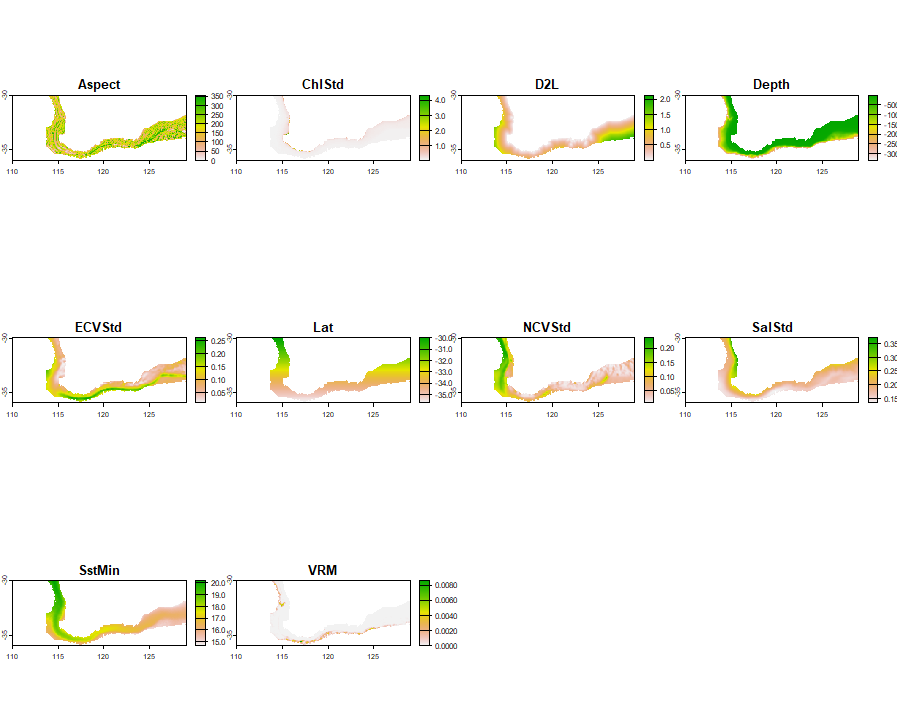

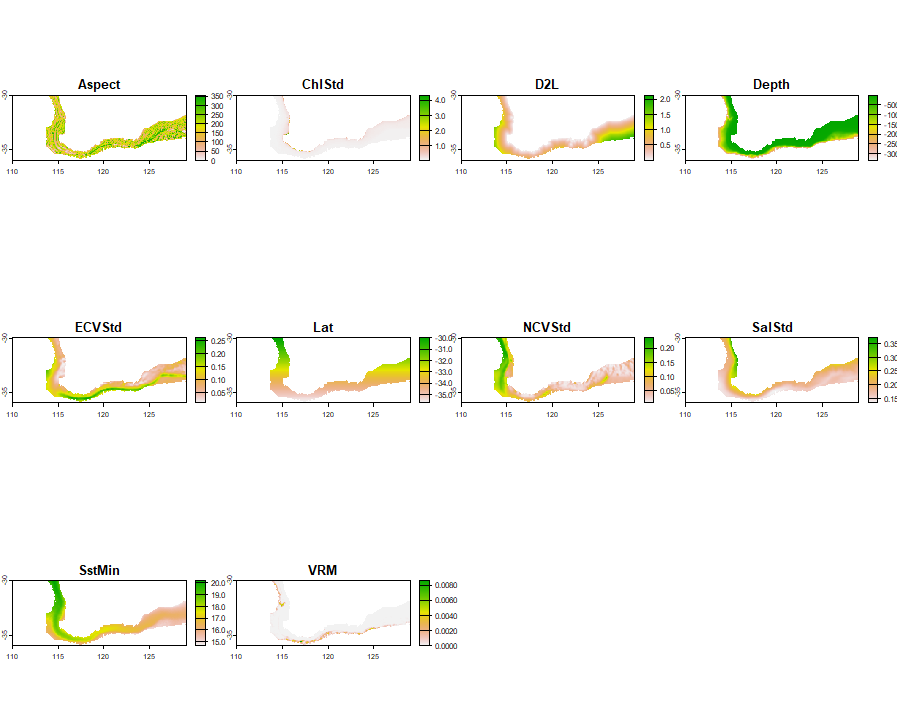

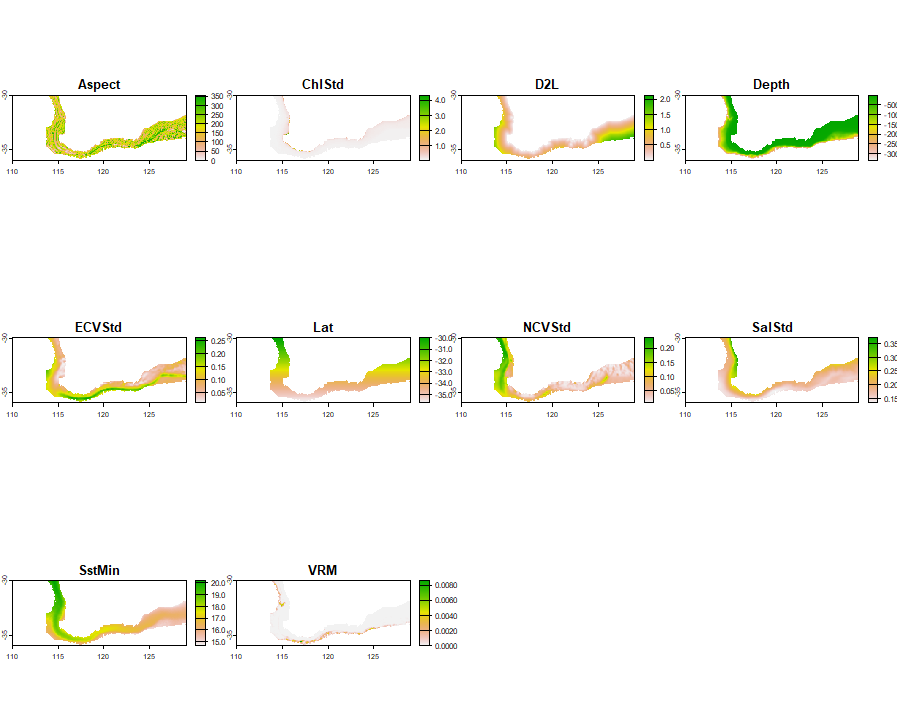

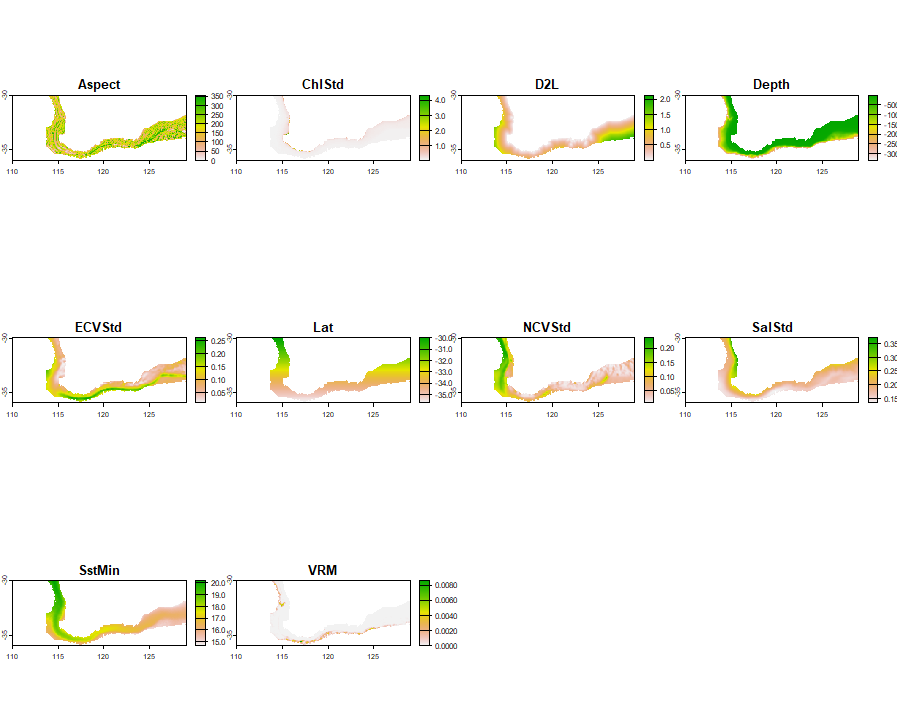


b)

c)


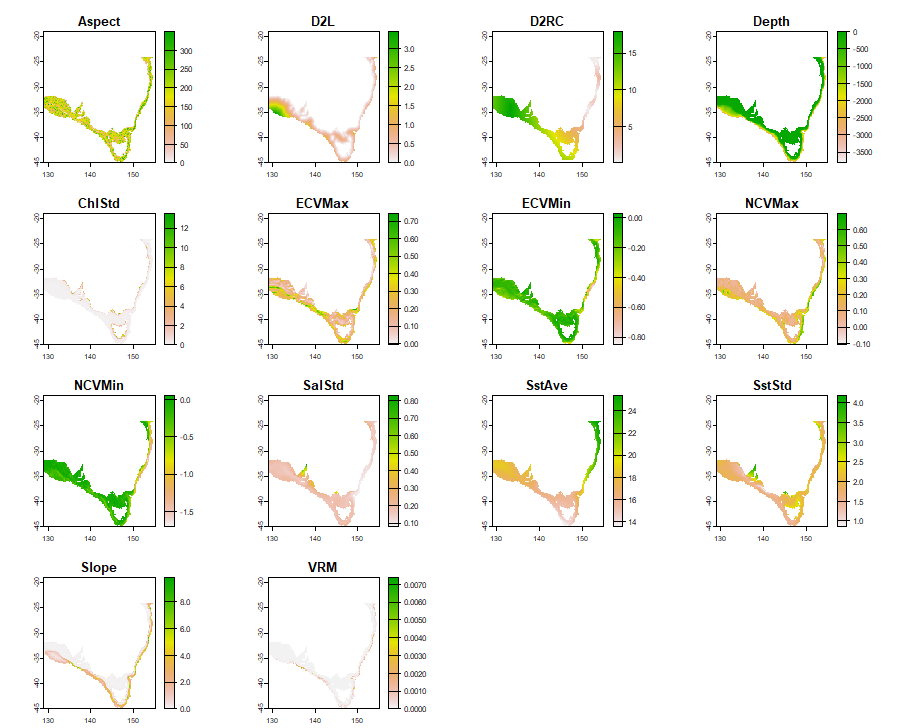

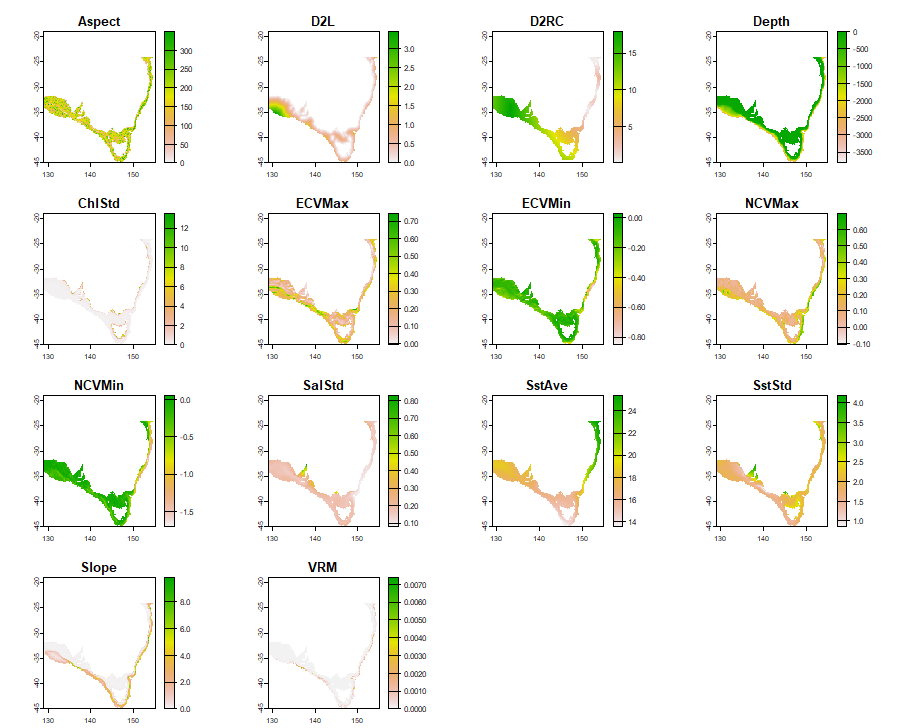

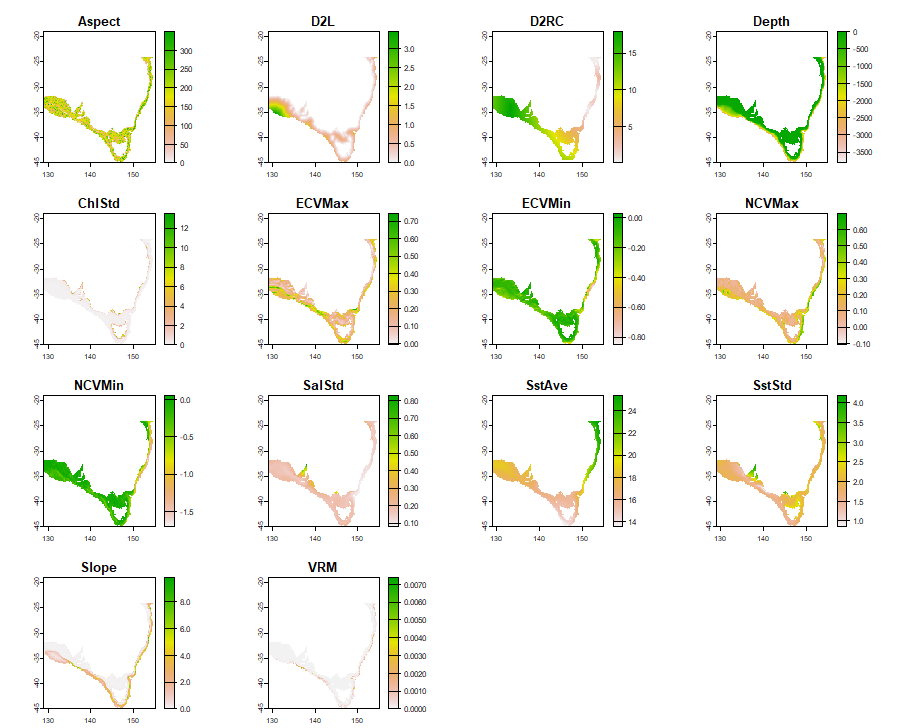


a)


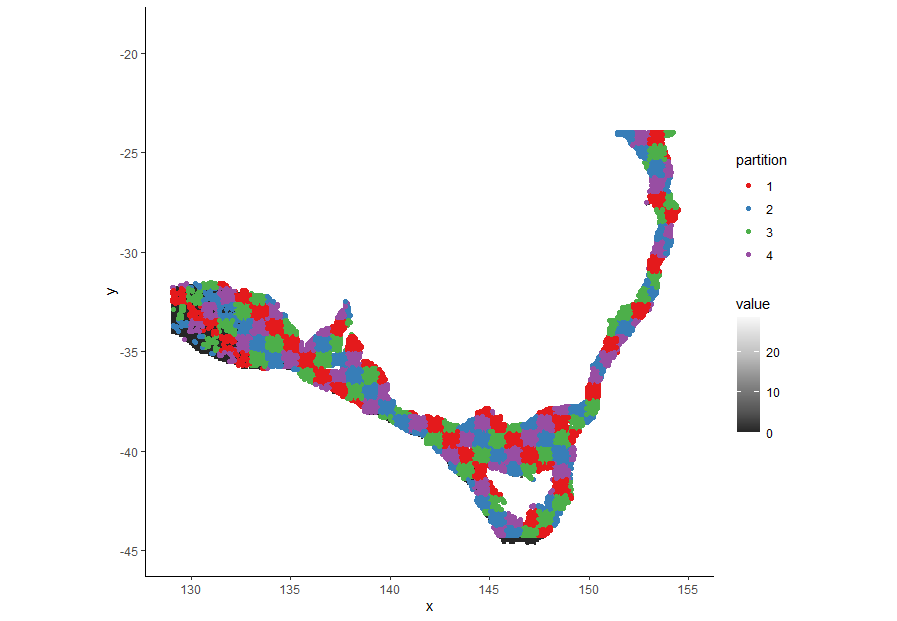

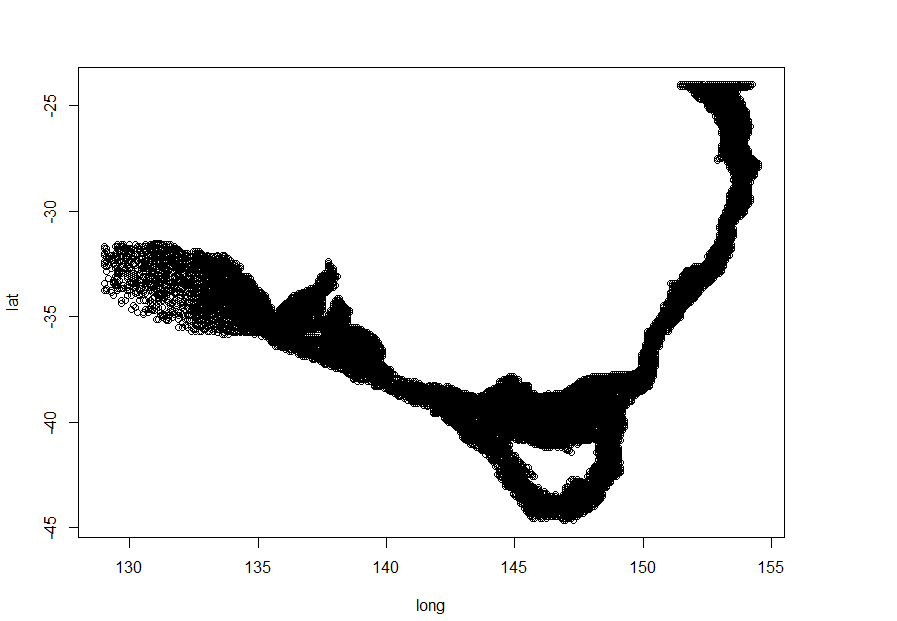

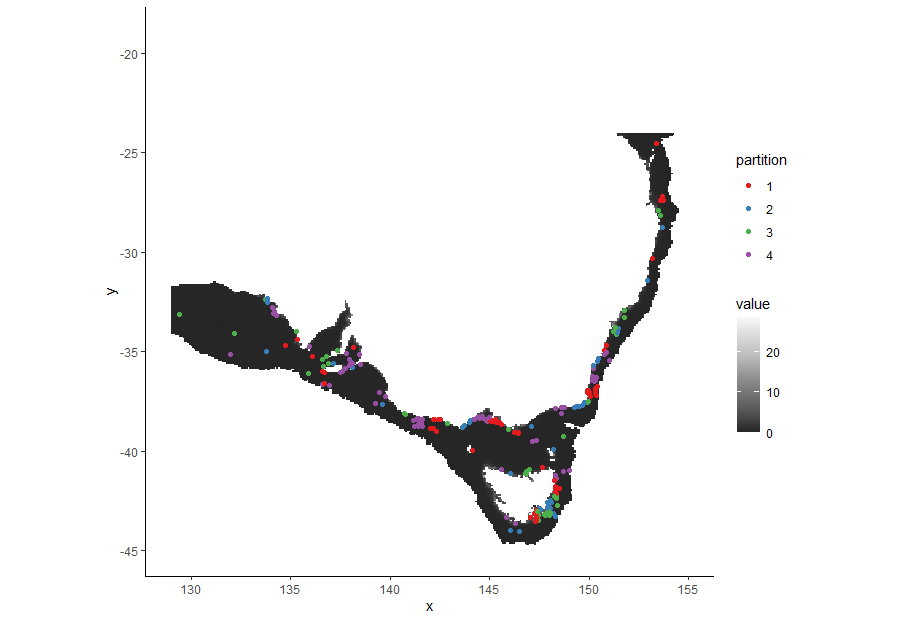
Appendix S6: a) Bias layer for the SDM of killer whales (*Orcinus orca*) in Australian waters of the southeast (SE) study area. b) Randomly selected background points for the SE study area. c) Spatially partitioned background points for the SE study area. d) Spatially partitioned presence points for the SE study area.

a)

d)

c)


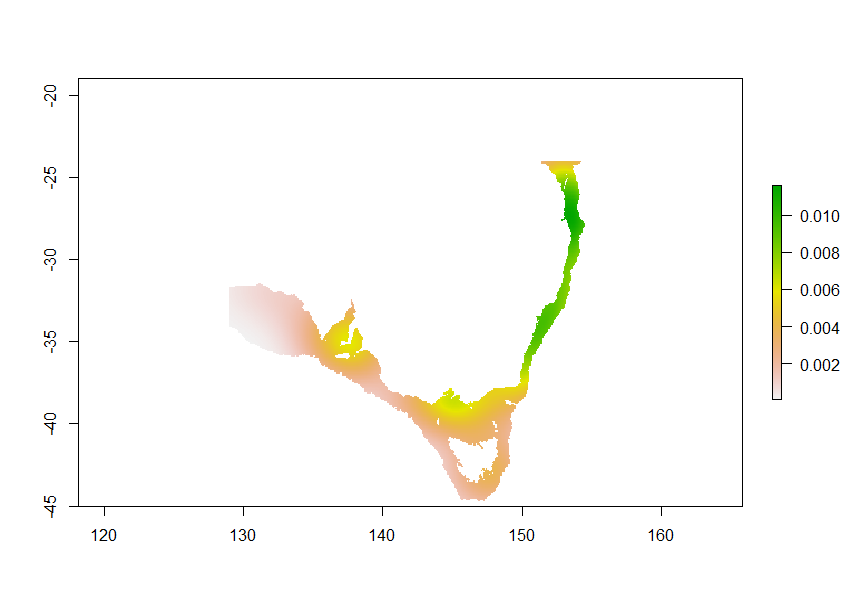


b)


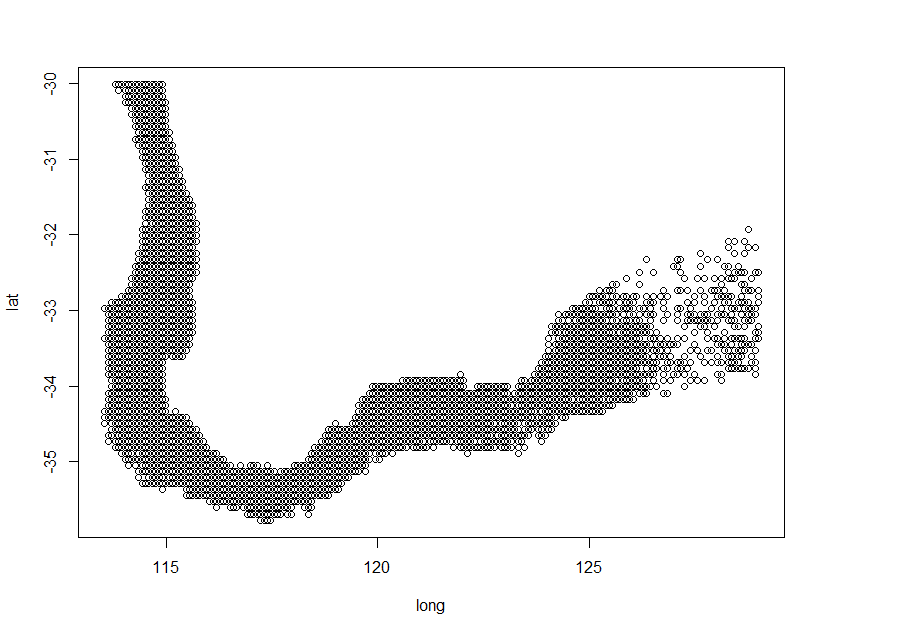
Appendix S7: a) Bias layer for the SDM of killer whales (*Orcinus orca*) in Australian waters of the southwest (SW) study area. b) Randomly selected background points for the SW study area. c) Jackknife partitioned background points for the SW study area. d) Jackknife partitioned presence points for the SW study area.


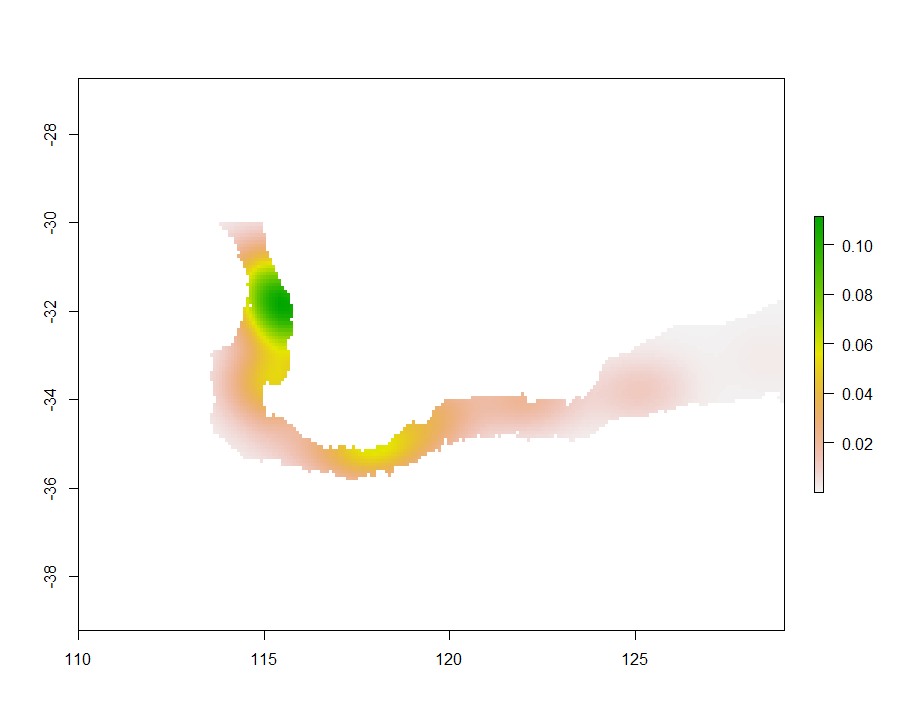


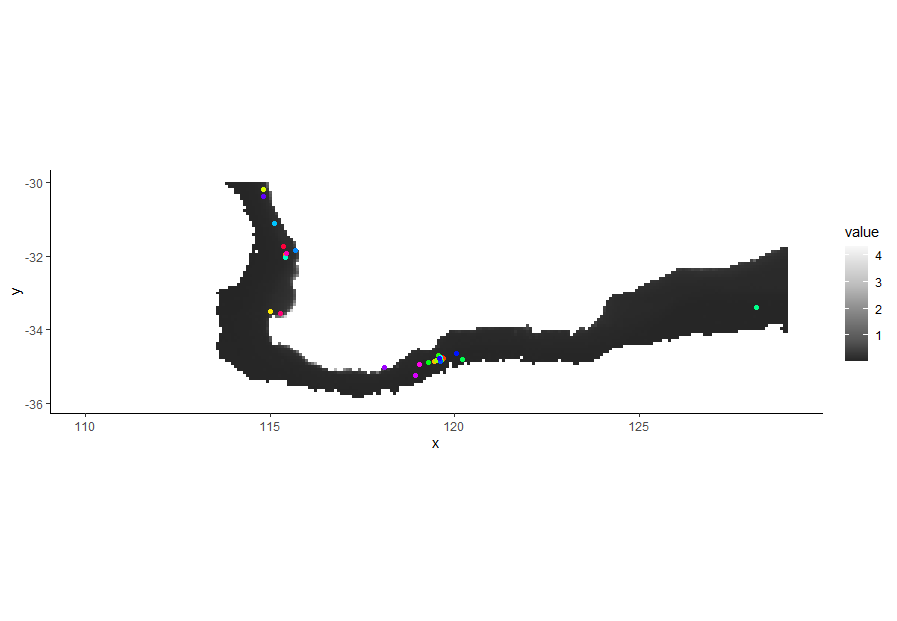

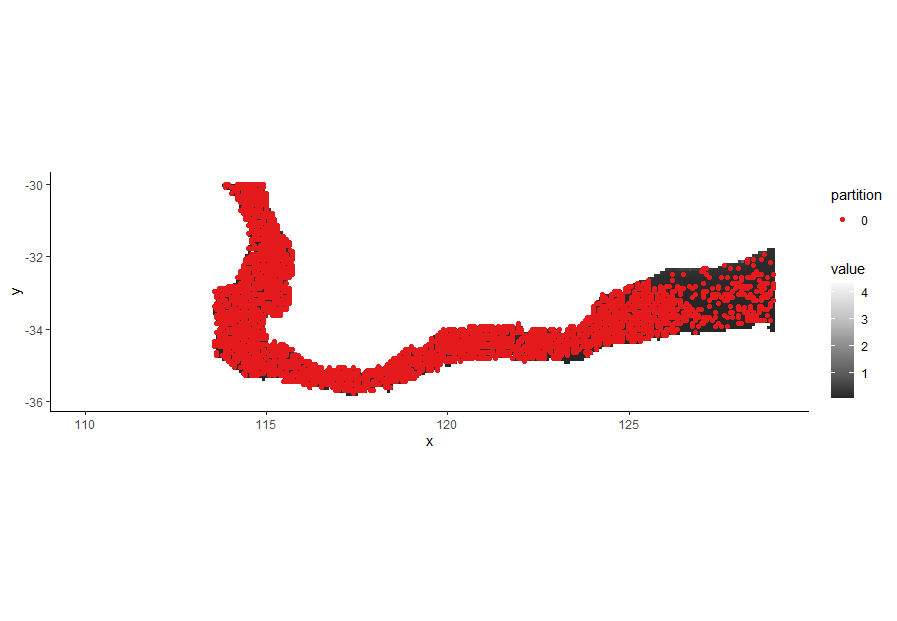


a)

b)

c)

d)


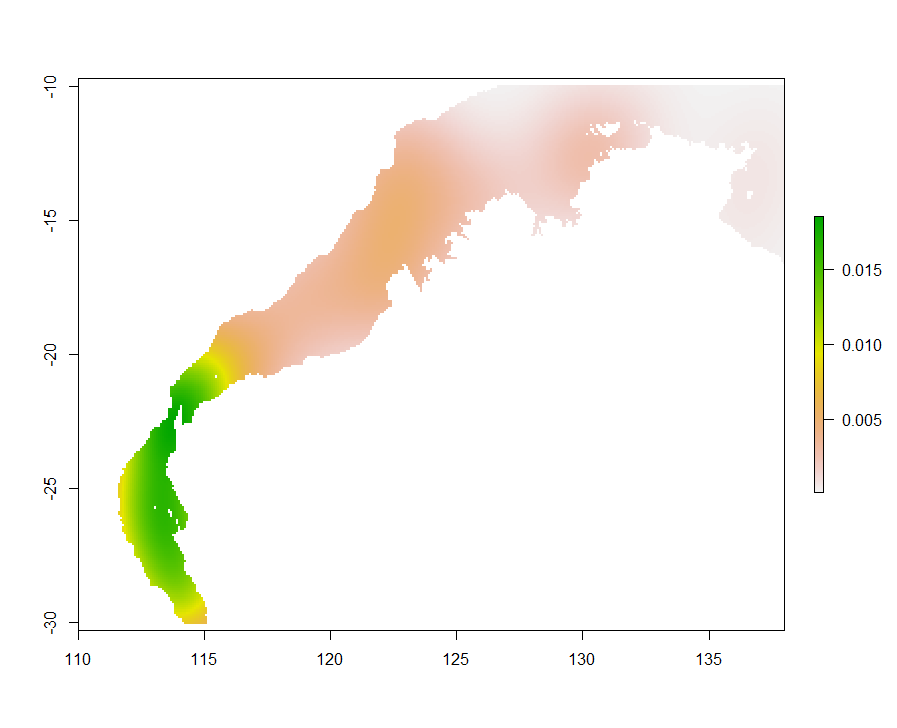

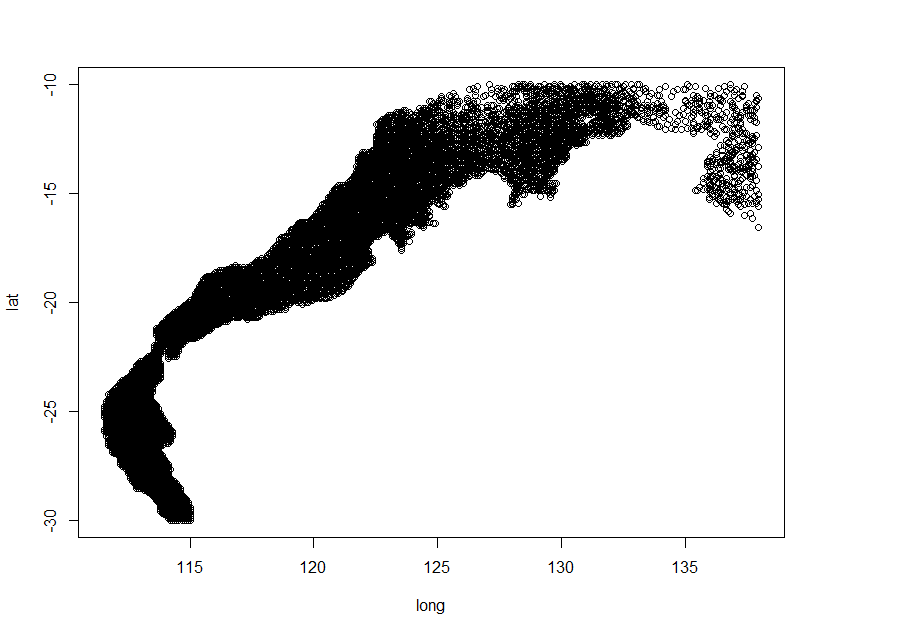

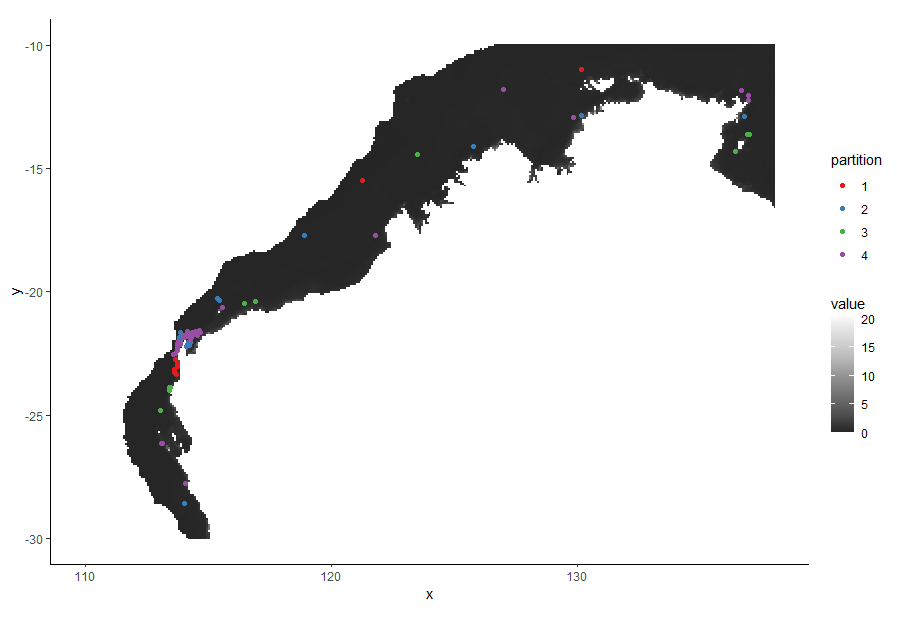

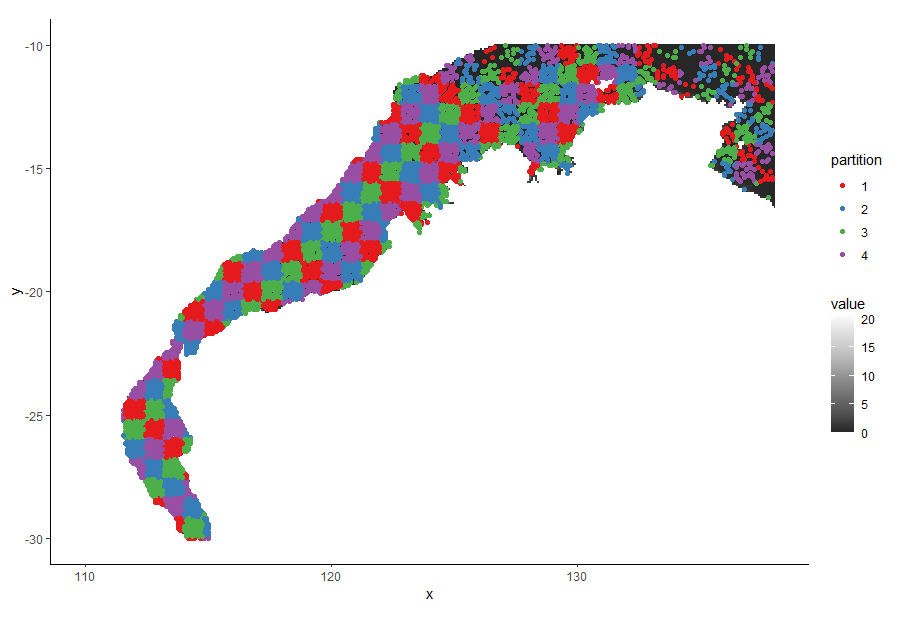
 Appendix S8: a) Bias layer for the SDM of killer whales (*Orcinus orca*) in Australian waters of the northwest (NW) study area. b) Randomly selected background points for the NW study area. c) Spatially partitioned background points for the NW study area. d) Spatially partitioned presence points for the NW study area.

a)

c)

b)

d)

Appendix S9: Univariate response curves of predictor variables used by MaxEnt for the SDM of killer whales (*Orcinus orca*) in Australian waters: a) of the southeast (SE) study area, b) of the southwest (SW) study area and c) of the northwest (NW) study area.


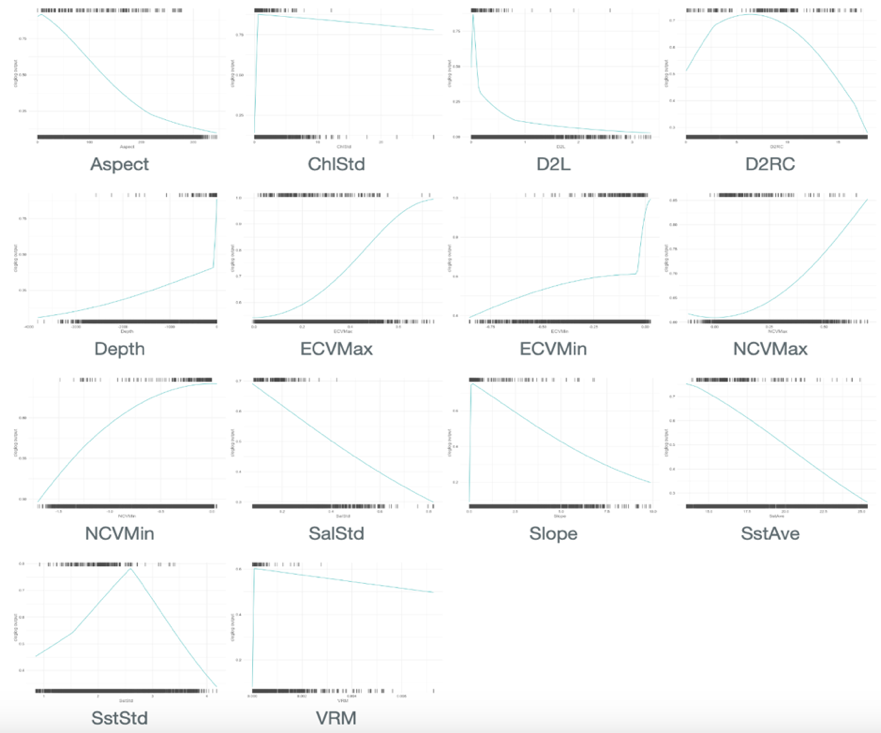

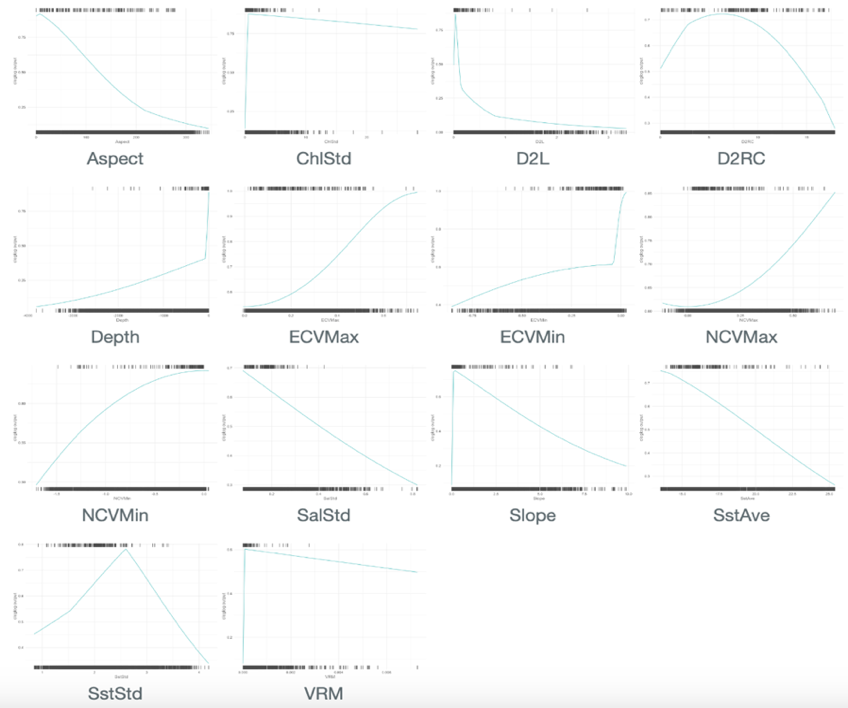

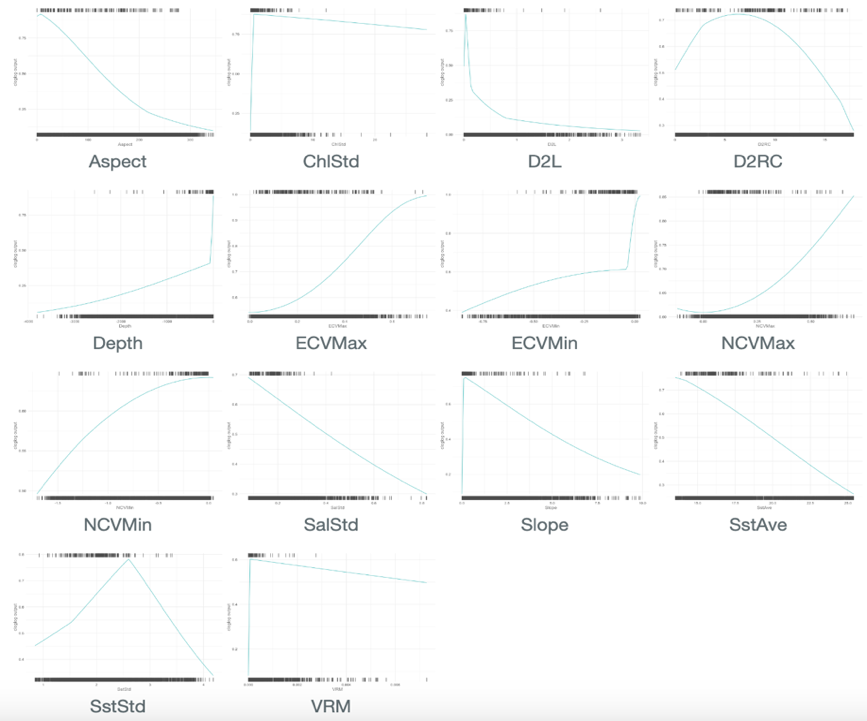


a)

b)


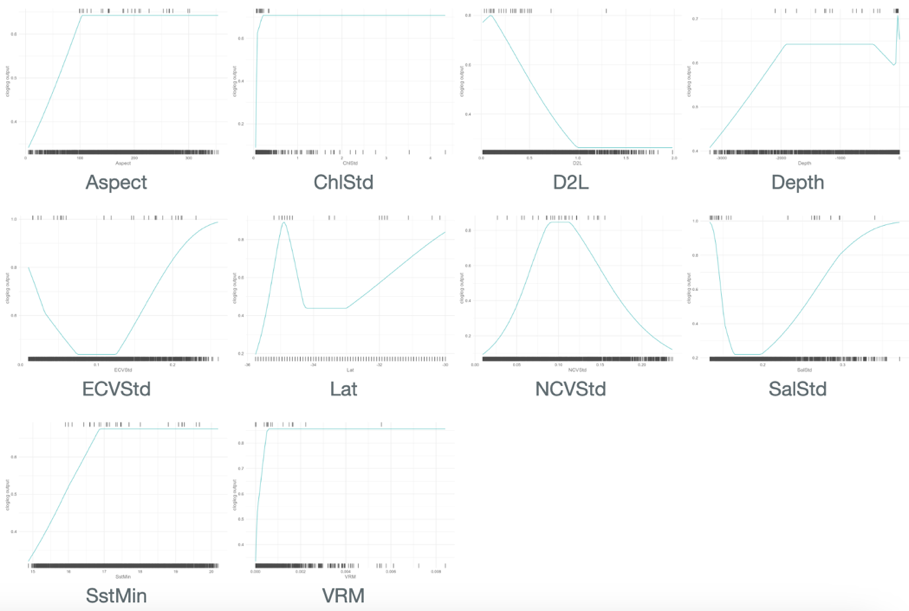

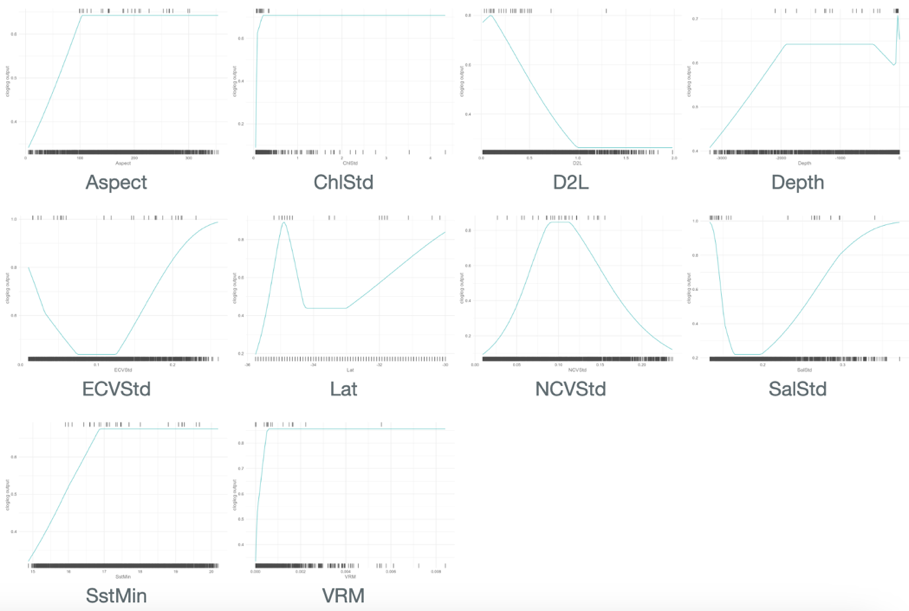


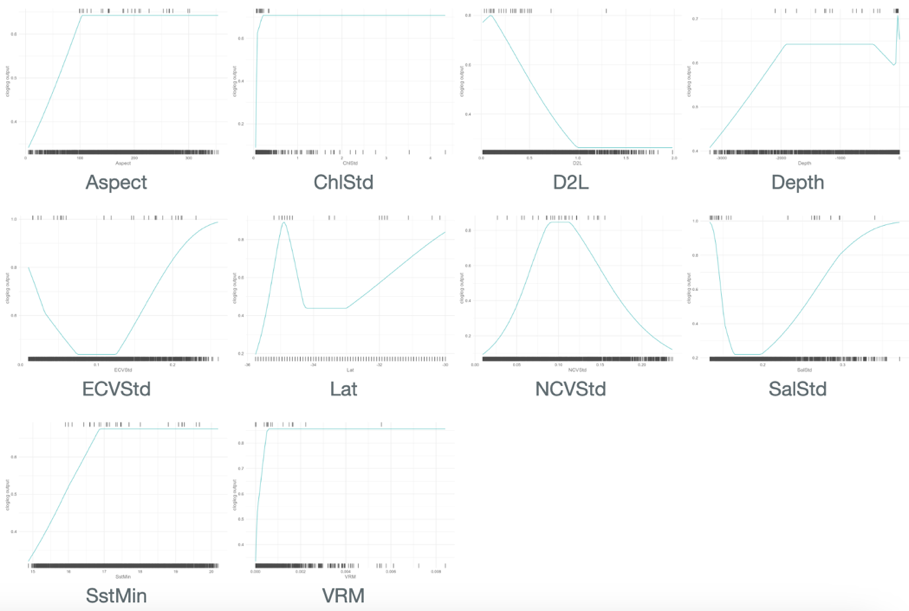


c)


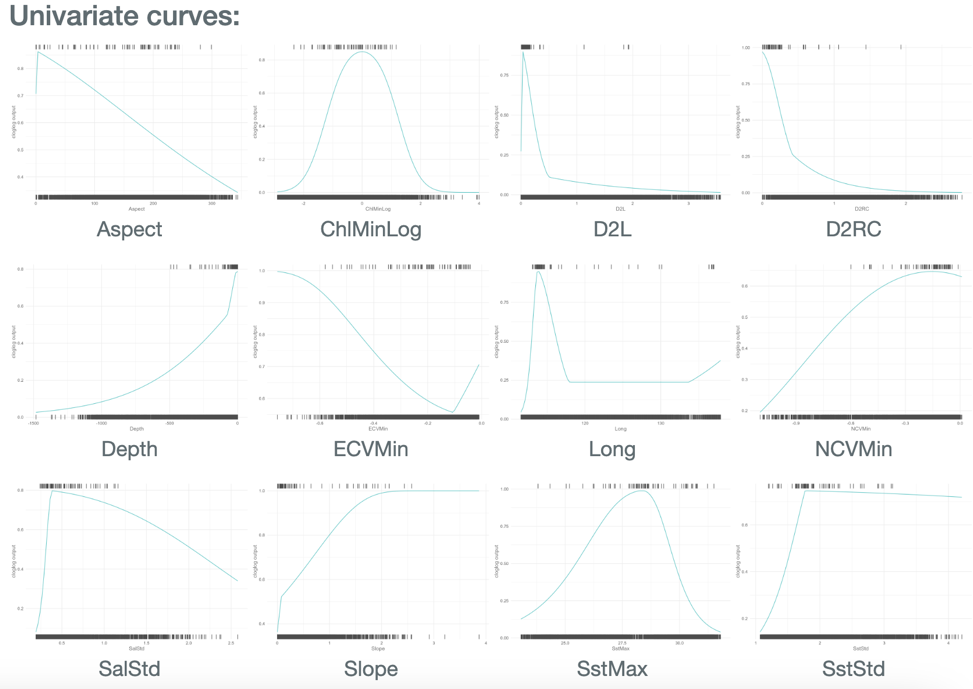


Appendix S10: a) ROC curve and AUC for the SDM of killer whales (*Orcinus orca*) in Australian waters: a) of the southeast (SE) study area, b) of the southwest (SW) study area and c) of the northwest (NW) study area.


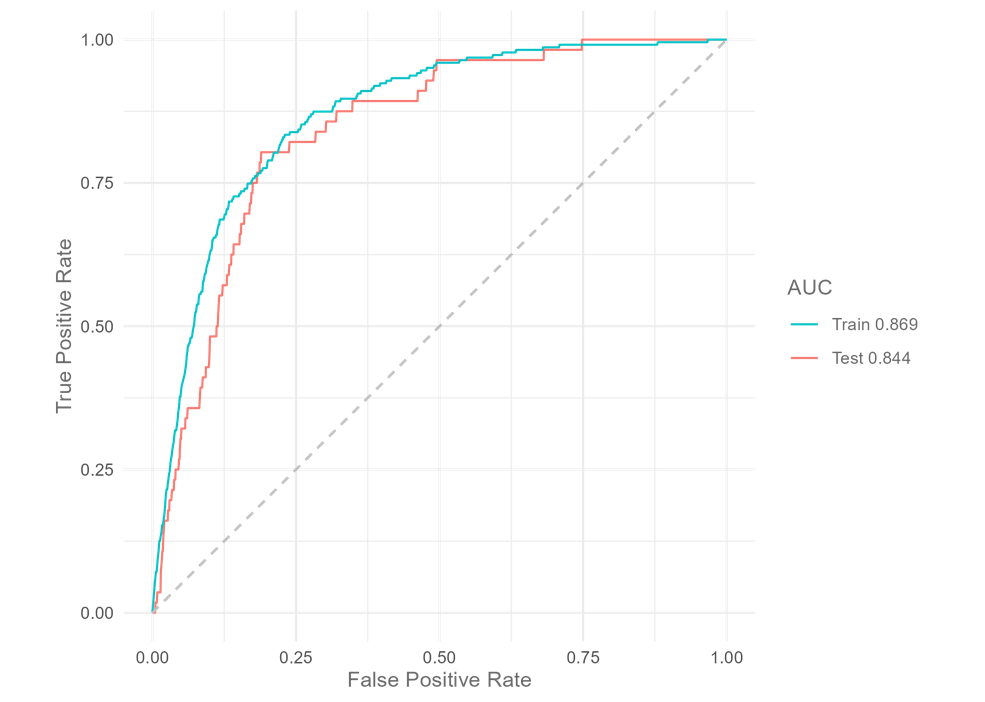

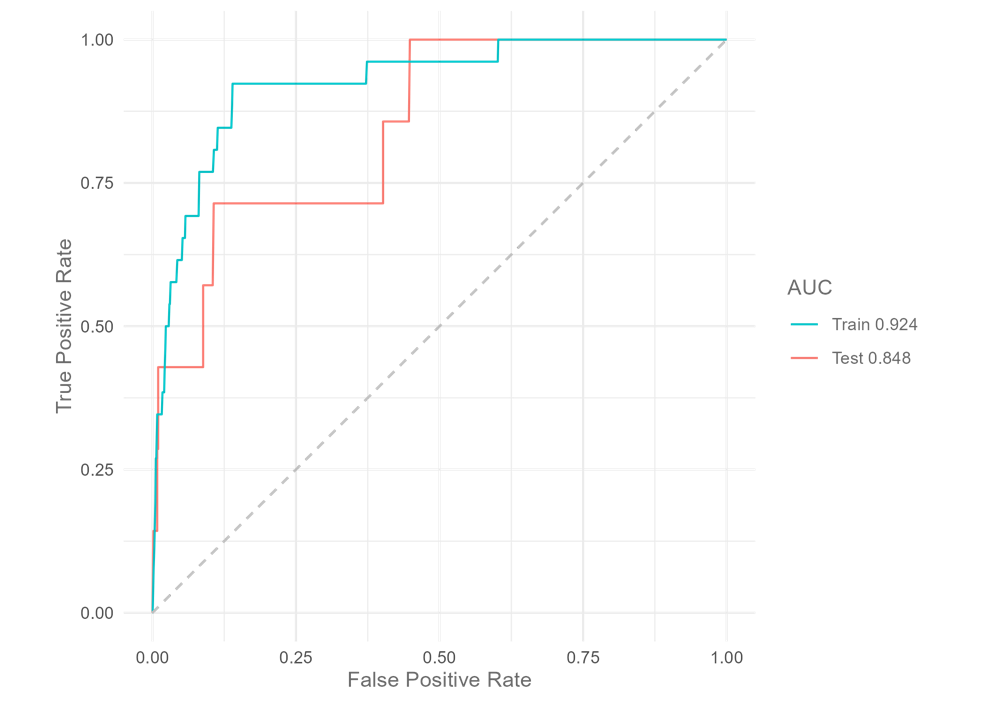

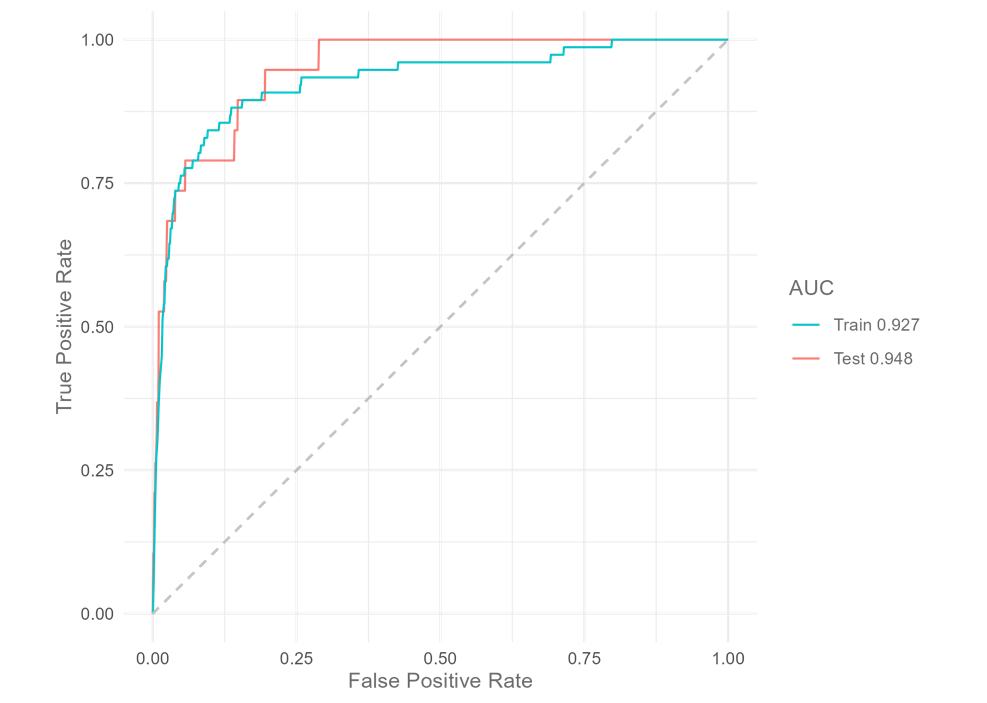


Appendix S11: ODMAP protocol for the SDM of killer whales (*Orcinus orca*) in Australian waters

c)

a)

b)

## Overview

#### Authorship

Contact: TBA

Study link: TBA

#### Model objective

Model objective: Forecast and transfer

Target output: Habitat suitability

#### Focal Taxon

Focal Taxon: Killer whale

#### Location

Location: Australia

#### Scale of Analysis

Spatial extent: 110, 155, -10, 45 (xmin, xmax, ymin, ymax)

Spatial resolution: 9.2 kms

Temporal extent: 1982-2023

Temporal resolution: Constant

Boundary: Natural

#### Biodiversity data

Observation type: Citizen science, field survey

Response data type: Presence-only, point occurrence

#### Predictors

Predictor types: Climatic, habitat

#### Hypotheses

Hypotheses: There is a tropical and temperate form of killer whale in Australia and the distribution of these animals is related to their environmental preferences

#### Assumptions

Model assumptions: This model is subject to sampling bias from a largely opportunistic dataset, but this was addressed as part of the spatial analysis.

#### Algorithms

Modelling techniques: MaxEnt

Model complexity: The model settings were chosen to yield simple, smooth response surfaces because we attempt extrapolation and anticipate violations of the critical model assumptions

Model averaging: Not performed

#### Workflow

Model workflow: Species occurrence data was compiled from a range of sources utilizing various sampling methods and manually cleaned to ensure reliability. Environmental data was downloaded from public online platforms. Some of these data were used to create subsequent rasters in ArcGIS Pro. RStudio was used to prepare spatial layers of predictor variables and bias layers of sampling effort. MaxEnt models were created in RStudio using SDMtune for three separate study areas. The steps employed were multi-collinearity testing of predictor variables, thinning of presence points, spatial partitioning of background and presence points, splitting data for training and testing by cross validation, model tuning, prediction, projection and evaluation

#### Software

Software: RStudio (v2023.12.1+402) with packages SDMtune and ENMeval

Code availability: Yes - please see Data Availability Statement of manuscript

Data availability: Yes - please see Data Availability Statement of manuscript

## Data

#### Biodiversity data

Taxon names: Killer whale

Taxonomic reference system: NA

Ecological level: Species

Data sources: Please see Appendix S1 of manuscript

Sampling design: Opportunistic

Sample size: 1115

Clipping: Nearshore Australian waters

Scaling: Spatial resolution of 9.2 kms within a constant temporal resolution

Cleaning: Species occurrence data was manually cleaned to filter out inconsistencies and duplicates

Absence data: Not available

Background data: A raster of targeted group survey effort was created to address sampling bias in each study area using the kernel density function in ArcGIS Pro. Sightings of any cetacean species which lied within the NW, SW and SE study areas were obtained from the Atlas of Living Australia. These had already been pre-screened for spatial bias, however they were further filtered to only those which were non killer whale, human observations, with imagery verification and submitted from the year 2000 onwards. Additional cetacean sightings were also sourced from the Centre for Whale Research (CWR) of Western Australia to increase the number of records in poorly sampled regions of the SW and NW study areas (e.g., offshore or remote locations). The randomPoints() function from the R package dismo was then used to generate the background points for which the density depended on the bias layer. The background points were thus subject to the same spatial bias as the presence points and fewer were placed in regions where there had been less survey effort. It was not necessary to limit the selection of background points to a buffer or polygon around the presence points due to the wide-ranging nature of the species and small size of the study areas. To reduce sampling bias in the presence points, spatial thinning was performed using the R package spThin so that only one sighting per grid cell was considered.

Errors and biases: There is potential for sampling bias from a largely opportunistic dataset

#### Data partitioning

Training data: 80% of the initial dataset was used for training

Validation data: The training data was spatially partitioned with the CheckerBoard2 or N-1 Jackknife options from the R package ENMeval

Test data: 20% of the initial dataset was kept out for testing

#### Predictor variables

Predictor variables: Distance to land (D2L), Distance to continental shelf break (D2CS), Distance to reef crest (D2RC), Depth, Latitude (Lat), Longitude (Long), Slope, Aspect, Vector ruggedness measure (VRM), Chlorophyll-a concentration (Chl - AveLog, MaxLog, MinLog and Std), Sea surface temperature (Sst - Ave, Max, Min and Std), Northward current velocity (NCV - Ave, Max, Min and Std), Eastward current velocity (ECV - Ave, Max, Min and Std) and Salinity (Sal - Ave, Max, Min and Std)

Data sources: IMOS, Geoscience Australia and Copernicus

Spatial extent: 110, 155, -10, -45 (xmin, xmax, ymin, ymax)

Spatial resolution: 9.2 kms

Coordinate reference system: WGS84

Temporal extent: Constant

Temporal resolution: 1992-2023

Data processing: All spatial layers were resampled to 9.2 kms through bilinear interpolation. A number of cell sizes were initially considered, but this was the value which best balanced the number of sightings, environmental data available, size of the study area and species dispersal ability. The Chl layers were also log transformed. Masking was used to set noninterest cells to not applicable (NA) for SDM. This was any which fell on land, as determined by a shapefile of the Australian coastline buffered by 0.04° (i.e., one half of a cell size) either side so that sightings that laid very close to this were not automatically discarded during modelling. Cells beyond a particular depth contour were also deemed offshore. This was any with a value greater than 1000 m for the NW and 2500 m for the SW and SE study areas. Some values along the coast had to be replaced by the mean of the surrounding cells to ensure all layers had the same number of NAs. The resulting study areas therefore only encompassed cells which fell between the shoreline and their respective depth contour.

Errors and biases: Unknown

Dimension reduction: An initial list of predictor variables was defined through consideration of those previously used in the literature and knowledge of the species.

#### Transfer data

Data sources: IMOS, Geoscience Australia and Copernicus

Spatial extent: 110, 155, -10, -45 (xmin, xmax, ymin, ymax)

Spatial resolution: 9.2 kms

Temporal extent: Constant

Temporal resolution: 1992-2023

Models and scenarios: Prediction to the whole of Australia

Data processing: All spatial layers were resampled to 0.008 degrees. The Chl layers were also log transformed.

Quantification of Novelty: Not undertaken

## Model

#### Variable pre-selection

Variable pre-selection: Final sets of predictor variables were determined through multi-colinearity testing and the varSel and reduceVar functions of the R package SDMtune on a default model. Those which were not correlated and maximized permutation importance were chosen in preference.

#### Multicollinearity

Multicollinearity: As above

#### Model settings

maxent: Model tuning was executed with the gridSearch() function from the R package SDMtune. Several variations of the regularisation multiplier (R: 1, 1.5, 2, 2.5, 3, 3.5, 4, 4.5 and 5) and feature class (FC: L, H, LH, LQ, LP, LQH, LQP, LQH, LQHP) were assessed to see how they influenced model complexity and fit. A total of 81 separate models were built on the training datasets of each study area using their cross-validation folds. The evaluation metric used to select the optimal settings was test TSS. The final model for each study area was then built using the function combineCV() so that the algorithm had access to all available training data.

Model settings (extrapolation): Clamping on

#### Model estimates

Coefficients: Mean

Parameter uncertainty: Cross validation during model tuning

Variable importance: Permutation importance and jackknifing on test TSS

#### Model selection - model averaging - ensembles

Model selection: test TSS

Model averaging: NA

Model ensembles: NA

#### Analysis and Correction of non-independence

Spatial autocorrelation: Spatial partitioning of both presence and background points was performed with the R package ENMeval. The CheckerBoard2 option with an aggregation factor of (10, 2) was used in the SE and NW study areas. This method creates four folds of which three are used to train the model and one is used to test thus cycling through four separate times. However, due to a smaller sample size for the SW study area, the N-1 jackknife option was used where each presence point is held out for testing in turn against all the other presence points. Background points are not partitioned in this case and models are built using all of them.

Temporal autocorrelation: NA

Nested data: NA

#### Threshold selection

Threshold selection: NA

## Assessment

#### Performance statistics

Performance on training data: TSS

Performance on validation data: TSS

Performance on test data: TSS, AUC

#### Plausibility check

Response shapes: Univariate response curves

Expert judgement: Map display

## Prediction

#### Prediction output

Prediction unit: The whole of Australia

Post-processing: NA

#### Uncertainty quantification

Algorithmic uncertainty: NA

Input data uncertainty: NA

Parameter uncertainty: NA

Scenario uncertainty: NA

Novel environments: Not undertaken
